# Opposite Roles of the JMJD1A Interaction Partners MDFI and MDFIC in Colorectal Cancer

**DOI:** 10.1038/s41598-020-65536-6

**Published:** 2020-05-26

**Authors:** Yuan Sui, Xiaomeng Li, Sangphil Oh, Bin Zhang, Willard M. Freeman, Sook Shin, Ralf Janknecht

**Affiliations:** 1 Department of Pathology, University of Oklahoma Health Sciences Center, Oklahoma City, OK 73104 USA; 2 China-Japan Union Hospital of Jilin University, Changchun, Jilin 130033 China; 3 Department of Cell Biology, University of Oklahoma Health Sciences Center, Oklahoma City, OK 73104 USA; 4 Stephenson Cancer Center, Oklahoma City, OK 73104 USA; 5 Genes and Human Disease Research Program, Oklahoma Medical Research Foundation, Oklahoma City, OK 73104 USA

**Keywords:** Colorectal cancer, Transcription

## Abstract

MyoD family inhibitor (MDFI) and MDFI domain-containing (MDFIC) are homologous proteins known to regulate myogenic transcription factors. Hitherto, their role in cancer is unknown. We discovered that *MDFI* is up- and *MDFIC* downregulated in colorectal tumors. Mirroring these different expression patterns, MDFI stimulated and MDFIC inhibited growth of HCT116 colorectal cancer cells. Further, MDFI and MDFIC interacted with Jumonji C domain-containing (JMJD) 1 A, a histone demethylase and epigenetic regulator involved in colorectal cancer. JMJD1A influenced transcription of several genes that were also regulated by MDFI or MDFIC. Notably, the *HIC1* tumor suppressor gene was stimulated by JMJD1A and MDFIC, but not by MDFI, and HIC1 overexpression phenocopied the growth suppressive effects of MDFIC in HCT116 cells. Similar to colorectal cancer, *MDFI* was up- and *MDFIC* downregulated in breast, ovarian and prostate cancer, but both were overexpressed in brain, gastric and pancreatic tumors that implies MDFIC to also promote tumorigenesis in certain tissues. Altogether, our data suggest a tumor modulating function for MDFI and MDFIC in colorectal and other cancers that may involve their interaction with JMJD1A and a MDFIC→HIC1 axis.

## Introduction

MyoD family inhibitor (MDFI; also known as I-mfa for inhibitor of MyoD family A) was originally cloned as an interaction partner of the MyoD family of myogenic transcription factors. MDFI represses their ability to activate gene transcription likely by two mechanisms: retention of these myogenic factors in the cytoplasm and inhibition of their nuclear DNA binding activity^1^. Consistently, MDFI is localized in both the cytoplasm and cell nuclei, albeit the predominant localization seems to be within the former^1,2^. Similar to its inhibitory impact on the MyoD family, MDFI binds to and suppresses the activity of the TCF/LEF-1 transcription factor that is a downstream effector in the WNT/β-catenin signaling pathway. While MDFI inhibits DNA binding of the *Xenopus* homolog XTcf3, it remains to be studied whether MDFI also diminishes the nuclear function of TCF/LEF-1 through sequestration within the cytoplasm^3–5^. In addition, MDFI binds to β-catenin and this interaction precludes MDFI from binding to MyoD family members, providing a mechanism by which WNT signaling, through increasing β-catenin levels, could overcome the inhibitory effects of MDFI on myogenesis^6^.

The biological function of MDFI was probed by homozygous deletion of its gene in mice. On a C57Bl/6 background, respective knockout mice die during embryogenesis, which is most likely due to a placental defect. However, on a 129/Sv background, *Mdfi*^*−/−*^ mice can survive into adulthood and be fertile; but various degrees of mild spina bifida and skeletal defects affecting the ribs were reported, with the most severe phenotypes causing death shortly after birth^7^. Another function of MDFI has been observed in osteoclasts: their number is increased and accordingly bone density reduced in *Mdfi*^*−/−*^ mice^8^. Furthermore, suppressing MDFI function through lentivirus-mediated downregulation promoted the regeneration of the murine gastrocnemius muscle after injury, possibly by increasing the activity of the MyoD and myogenin transcription factors^9^.

A homolog of MDFI is MyoD family inhibitor domain-containing (MDFIC), which also preferentially localizes within the cytoplasm. However, a rare longer MDFIC isoform localizes around and in nucleoli^10,11^. This isoform may be important to interact with and sequester the HAND1 transcription factor within nucleoli, which is predicted to suppress HAND1-dependent placentation and cardiac morphogenesis^12^. MDFIC also binds to the glucocorticoid receptor in the cytoplasm, which leads to a change in glucocorticoid receptor phosphorylation. When cells were treated with glucocorticoid, this interaction dissolved and the receptor translocated into the cell nucleus while MDFIC stayed behind in the cytoplasm. Moreover, transcriptome analyses revealed that MDFIC can influence the inflammatory response mediated by the glucocorticoid receptor^13^. However, no *Mdfic*^*-/-*^ mouse model has yet been published that could corroborate these potential functions of MDFIC.

Presently, it is essentially unknown whether MDFI and MDFIC play any role in tumor formation. We found that MDFI and MDFIC are capable of interacting with JMJD1A, a member of the Jumonji C domain-containing (JMJD) protein family. JMJD1A, also called lysine demethylase 3 A (KDM3A), can demethylate di- and monomethylated lysine 9 on histone H3^14^ and may exert pro-oncogenic functions in colon cancer cells^15–19^. In addition, we discovered changes in the expression pattern of *MDFI* and *MDFIC* in colorectal tumors. Hence, we examined the role of MDFI and MDFIC in colorectal cancer cells.

## Results

### Binding of MDFI and MDFIC to JMJD1A

**Figure 1:**
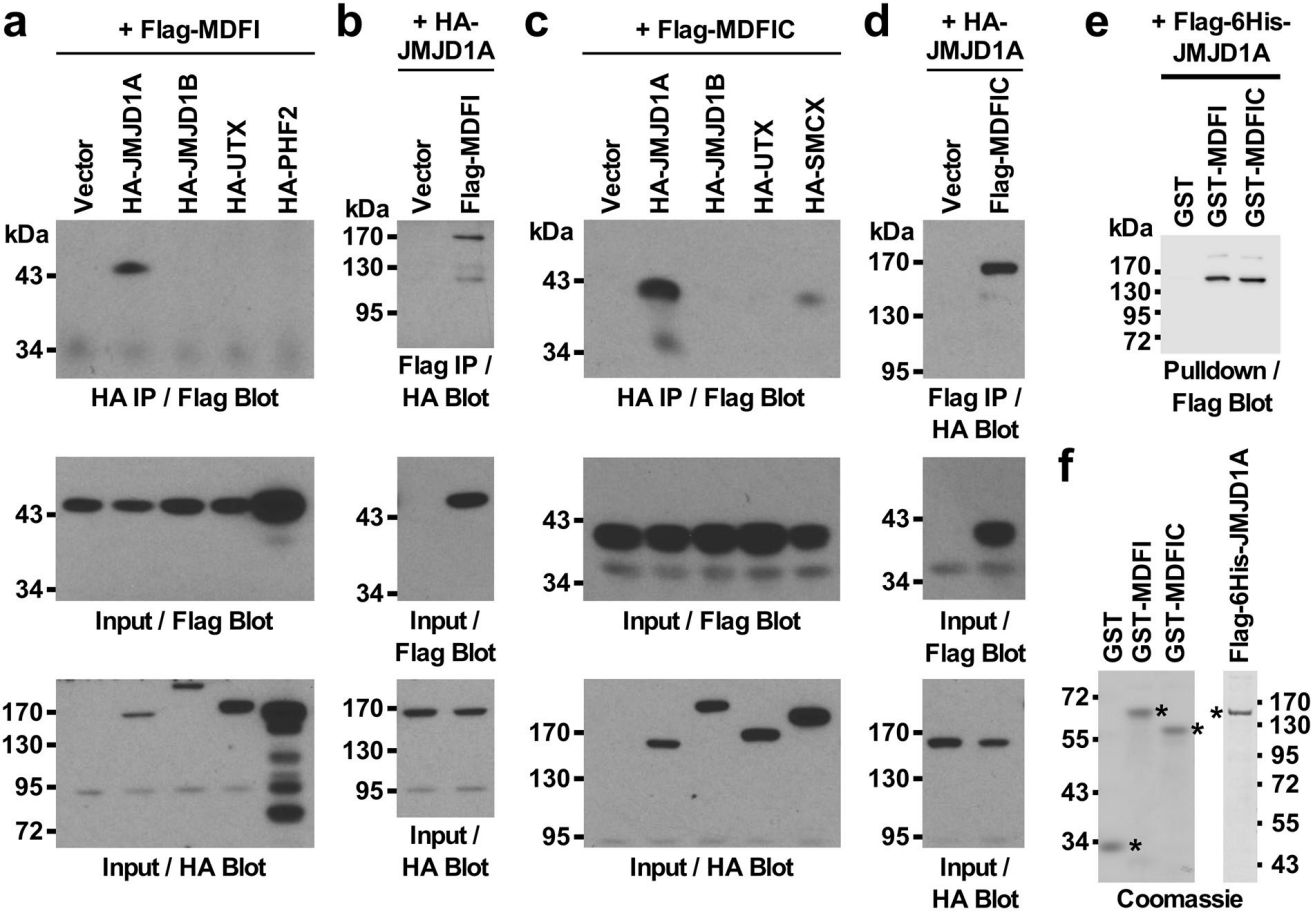
Interaction of JMJD1A with MDFI and MDFIC. (**a**) Flag-tagged MDFI was coexpressed with indicated HA-tagged JMJD proteins (JMJD1A, JMJD1B, UTX or PHF2). After anti-HA immunoprecipitation (IP), coprecipitated MDFI was detected by anti-Flag blotting (top panel). The bottom two panels show input levels of Flag- or HA-tagged proteins. (**b**) Respective reverse order coimmunoprecipitation experiment: anti-Flag IP followed by anti-HA blotting. (**c**) Coimmunoprecipitation experiments with Flag-MDFIC and HA-tagged JMJD1A, JMJD1B, UTX or SMCX. (**d**) Corresponding reverse order coimmunoprecipitation experiment with Flag-MDFIC and HA-JMJD1A. (**e**) Binding of purified, Flag-6His-JMJD1A to comparable amounts of purified GST, GST-MDFI or GST-MDFIC. (**f**) Coomassie-stained protein gels revealing the purity of respective recombinant proteins. Full-size blots are presented in Supplementary Figs. S1 and S2b,c.

Pursuing our long-standing interest in the histone demethylase JMJD1A and its interactome^20^, we also tested if JMJD1A might interact with MDFI or MDFIC. To this end, we performed coimmunoprecipitation experiments. When Flag-tagged MDFI was coexpressed with HA-tagged JMJD1A, MDFI coprecipitated with JMJD1A, but not with the homologous JMJD1B or two other JMJD proteins, UTX and PHF2 (Fig. 1a and Supplementary Fig. S1a). This complex formation between MDFI and JMJD1A was also observable in a reverse order coimmunoprecipitation experiment (Fig. 1b and Supplementary Fig. S1b). Likewise, complex formation was noted between MDFIC and JMJD1A (Fig. 1c,d and Supplementary Fig. S1c,d). Furthermore, when comparable amounts of MDFI and MDFIC were expressed, their degree of complex formation with JMJD1A was similar (Supplementary Fig. S2a).

To determine whether JMJD1A binds directly to MDFI and MDFIC, we purified respective proteins and tested their interaction *in vitro*. Indeed, purified JMJD1A bound to purified MDFI and MDFIC (Fig. 1e,f and Supplementary Fig. S2b,c). Lastly, we observed that MDFI and MDFIC did not interact with the conserved catalytic center of JMJD1A, possibly explaining why not all JMJD proteins are interaction partners of MDFI/MDFIC, while the highly homologous, cysteine-rich C-termini of MDFI and MDFIC were responsible for binding to JMJD1A (Supplementary Fig. S3). Altogether, we conclude that MDFI and MDFIC can directly bind to the histone demethylase JMJD1A.

### Opposite changes of MDFI and MDFIC expression in colorectal cancer

**Figure 2:**
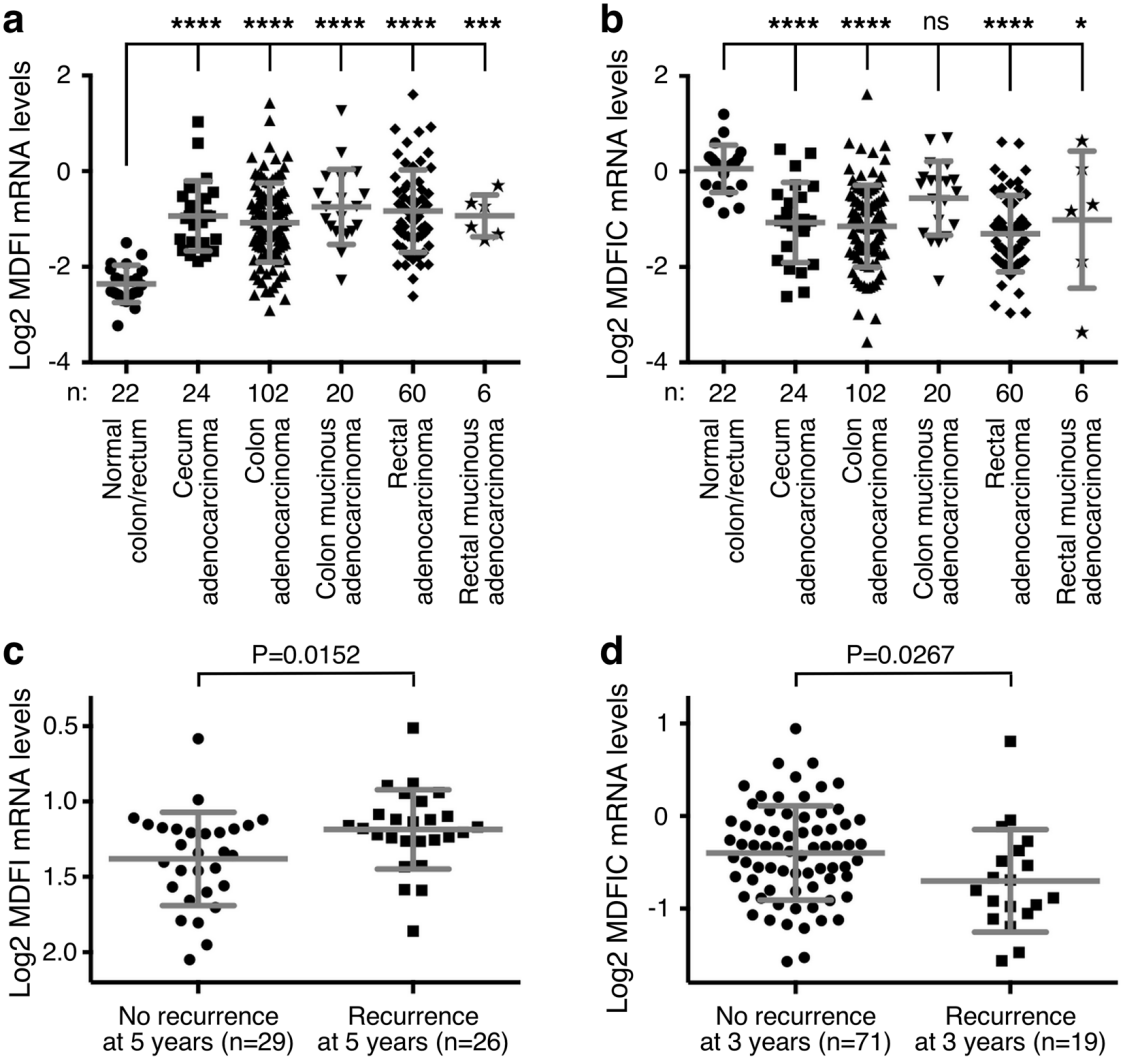
Altered expression of *MDFI* and *MDFIC* in colorectal tumors. (**a**) *MDFI* or (**b**) *MDFIC* mRNA levels in normal and diseased colorectal tissues. Data were derived from TCGA microarray experiments (reporter A_23_P42165 for *MDFI* and A_23_P327020 for *MDFIC*). One-way ANOVA (Dunnett’s multiple comparisons test): *P < 0.05; ***P < 0.001; ****P < 0.0001; ns, not significant. (**c**) Association of *MDFI* (reporter 205375_at; data from Lin *et al*.^22^) or (**d**) *MDFIC* (reporter 1559942_at; data from Jorissen *et al*.^23^) mRNA levels with recurrence of colorectal carcinomas; unpaired, two-tailed t test. In all four panels, means with standard deviations are shown.

Because JMJD1A is overexpressed in colorectal cancer^16–19^ and physically interacts with MDFI/MDFIC, we assessed potential changes of *MDFI* and *MDFIC* expression in colorectal tumors by analyzing published microarray data with Oncomine (www.oncomine.org). The Cancer Genome Atlas (TCGA) data set^21^ revealed that *MDFI* mRNA was significantly upregulated in cecum, colon, colon mucinous, rectal and rectal mucinous adenocarcinomas compared to normal colon and rectum (Fig. 2a), while *MDFIC* was on average downregulated (Fig. 2b). Similar *MDFI* up- and *MDFIC* downregulation was observed in other microarray data sets, and *MDFI* appears to be also more expressed in metastatic compared to primary tumor sites (Supplementary Fig. S4). Furthermore, high *MDFI* mRNA levels were associated with recurrence of colorectal cancer (Fig. 2c; data from reference^22^), while high *MDFIC* levels were linked to absence of recurrence (Fig. 2d; data from reference^23^). Together, these data suggest that *MDFI* up- and *MDFIC* downregulation are connected to colorectal tumor formation and possibly also the aggressiveness of the disease.

### Impact of MDFI and MDFIC overexpression on HCT116 cells

**Figure 3:**
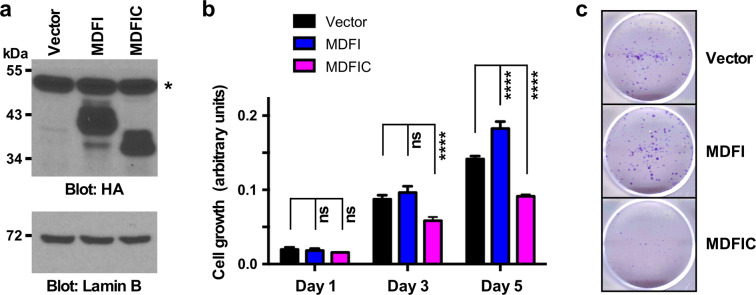
Impact of MDFI and MDFIC on cell growth. (**a**) HCT116 colorectal cancer cells were transduced with retrovirus encoding HA-tagged MDFI or MDFIC. Shown are anti-HA and anti-Lamin B blots. Asterisk marks an unspecific band. Uncropped Western blots are presented in Supplementary Fig. S5a. (**b**) Growth assays. Two-way ANOVA (Dunnett’s multiple comparison tests); shown are means with standard deviations (n = 3). ****P < 0.0001; ns, not significant. (**c**) Representative clonogenic assay.

To assess if MDFI or MDFIC can affect cancer cells, we overexpressed these proteins in human HCT116 colorectal cancer cells (Fig. 3a and Supplementary Fig. S5a) and then examined their growth. Of note, MDFI overexpression led to increased cell growth, whereas MDFIC reduced it (Fig. 3b). Moreover, while MDFI overexpression did not robustly affect clonogenic activity, MDFIC strongly suppressed it (Fig. 3c). These results are consistent with the notion that MDFI may promote while MDFIC may inhibit colon cancer formation, which highlights stark differences in function between MDFI and MDFIC despite their homologous amino acid sequences.

To complement the above overexpression experiments, we also downregulated MDFI or MDFIC with two different shRNAs in HCT116 cells (Supplementary Fig. S6a,d). This did not cause any significant changes in HCT116 cell growth (Supplementary Fig. S6b,e) and also did not affect clonogenic activity (Supplementary Fig. S6c,f). Unfortunately, the unavailability of high-affinity and specific anti-MDFI and anti-MDFIC antibodies did not allow us to measure endogenous MDFI and MDFIC protein levels. If they were very low, any downregulation, although detectable by RT-PCR as shown in Supplementary Fig. S6a,d, would be expected to have no observable impact on HCT116 cells. As such, the results of the shown RNA interference experiments do not allow us to strengthen or refute the hypothesis that MDFI exerts tumor-promoting and MDFIC tumor-suppressing activities.

### Transcriptome analysis

**Figure 4:**
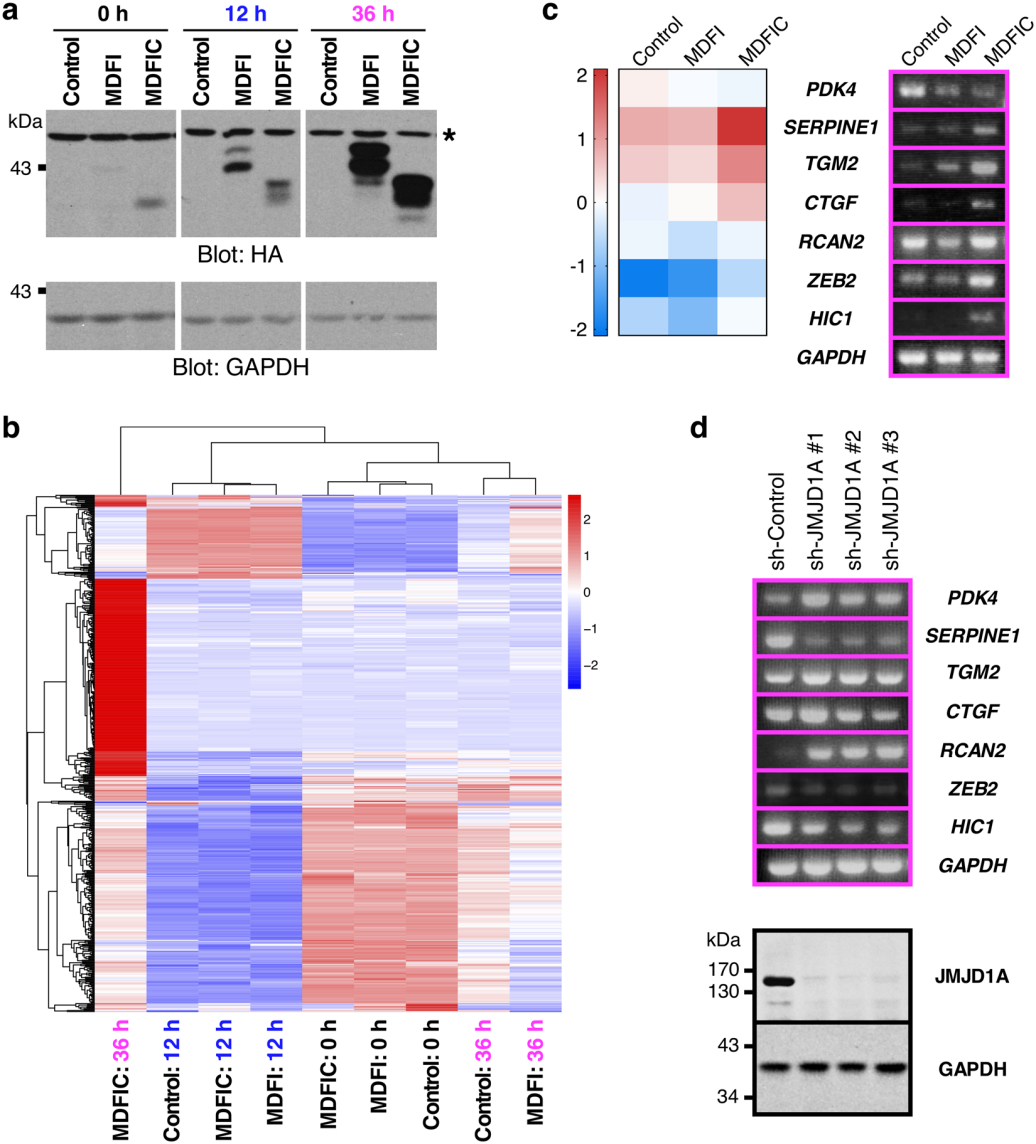
Transcriptome analysis in HCT116 cells. (**a**) Doxycycline-mediated upregulation of HA-tagged MDFI and MDFIC after 12 and 36 h. Asterisk marks an unspecific band recognized by the anti-HA antibody. Uncropped Western blots are presented in Supplementary Fig. S5b. (**b**) Hierarchical clustering of 525 differentially expressed genes. (**c**) Validation of RNA sequencing data (left panel) by RT-PCR (right panel) for indicated target genes after 36 h of doxycycline induction; *GAPDH* served as a control. Uncropped agarose gels are shown in Supplementary Fig. S5c. (**d**) Downregulation of JMJD1A with three different shRNAs. Shown are RT-PCR results (top) and Western blots (bottom). Uncropped agarose gels are shown in Supplementary Fig. S9a, and uncropped Western blots in Supplementary Fig. S9b.

To gain more insights into a transcriptional role of MDFI and MDFIC, we created doxycycline-inducible HCT116 cells overexpressing these two proteins. Robust induction of MDFI and MDFIC was observed 12 h after doxycycline addition, and even more so after 36 h (Fig. 4a and Supplementary Fig. S5b). RNA sequencing and cluster analysis of differentially expressed genes revealed that only MDFIC-overexpressing cells were starkly different after 36 h of doxycycline treatment (Fig. 4b and Supplementary Fig. S7). Comparison of RNA sequencing results with RT-PCR data for seven selected genes showed principally consistent trends (Fig. 4c and Supplementary Fig. S5c), thereby validating our RNA sequencing results. This also again emphasized differences between MDFI and MDFIC. For instance, *RCAN2* was downregulated upon MDFI overexpression, but not by MDFIC. Or *SERPINE1*, *CTGF*, *ZEB2* and *HIC1* were upregulated by MDFIC, but not by MDFI.

We wondered if a differential intracellular localization could be responsible for this different behavior of MDFI and MDFIC. However, MDFI and MDFIC displayed a similar intracellular distribution in HCT116 cells (Supplementary Fig. S8), with both proteins being predominantly in the cytoplasm which is comparable to previously observed data in mouse NIH3T3 fibroblasts or African green monkey COS-1 or COS-7 kidney fibroblast-like cells^1,2,10,11^. But please note that a small fraction of MDFI and MDFIC was also present in cell nuclei and the insoluble fraction consisting primarily of chromatin, indicating that MDFI and MDFIC are in principle capable of acting as nuclear transcriptional cofactors in HCT116 cells.

We then examined if the above mentioned seven target genes would also be regulated by JMJD1A. To this end, we downregulated JMJD1A with three different shRNAs that led to efficient reduction of JMJD1A protein levels (Fig. 4d, bottom panels, and Supplementary Fig. S9b). With the exception of *CTGF*, all three JMJD1A shRNAs led to consistent changes in mRNA levels (Fig. 4d, top panels, and Supplementary Fig. S9a), suggesting that *PDK4*, *SERPINE1*, *TGM2*, *RCAN2*, *ZEB2* and *HIC1* are JMJD1A target genes. Interestingly, while MDFIC downregulated *PDK4*, JMJD1A shRNA caused upregulation, and when MDFIC caused upregulation of mRNA levels (*SERPINE1*, *ZEB2*, *HIC1*), JMJD1A depletion had the opposite effect; this suggests that MDFIC and JMJD1A cooperate in the regulation of *PDK4*, *SERPINE1*, *ZEB2* and *HIC1* transcription. Likewise, *RCAN2* transcription might be cooperatively repressed by MDFI and JMJD1A. In contrast, JMJD1A downregulation as well as MDFIC overexpression led to *TGM2* upregulation, implying antagonistic roles for MDFIC and JMJD1A in *TGM2* transcriptional regulation. Overall, these data implicate that JMJD1A can potentially impinge on the transcriptional effects of MDFI and MDFIC.

### HIC1 as a potential downstream effector of MDFIC

**Figure 5:**
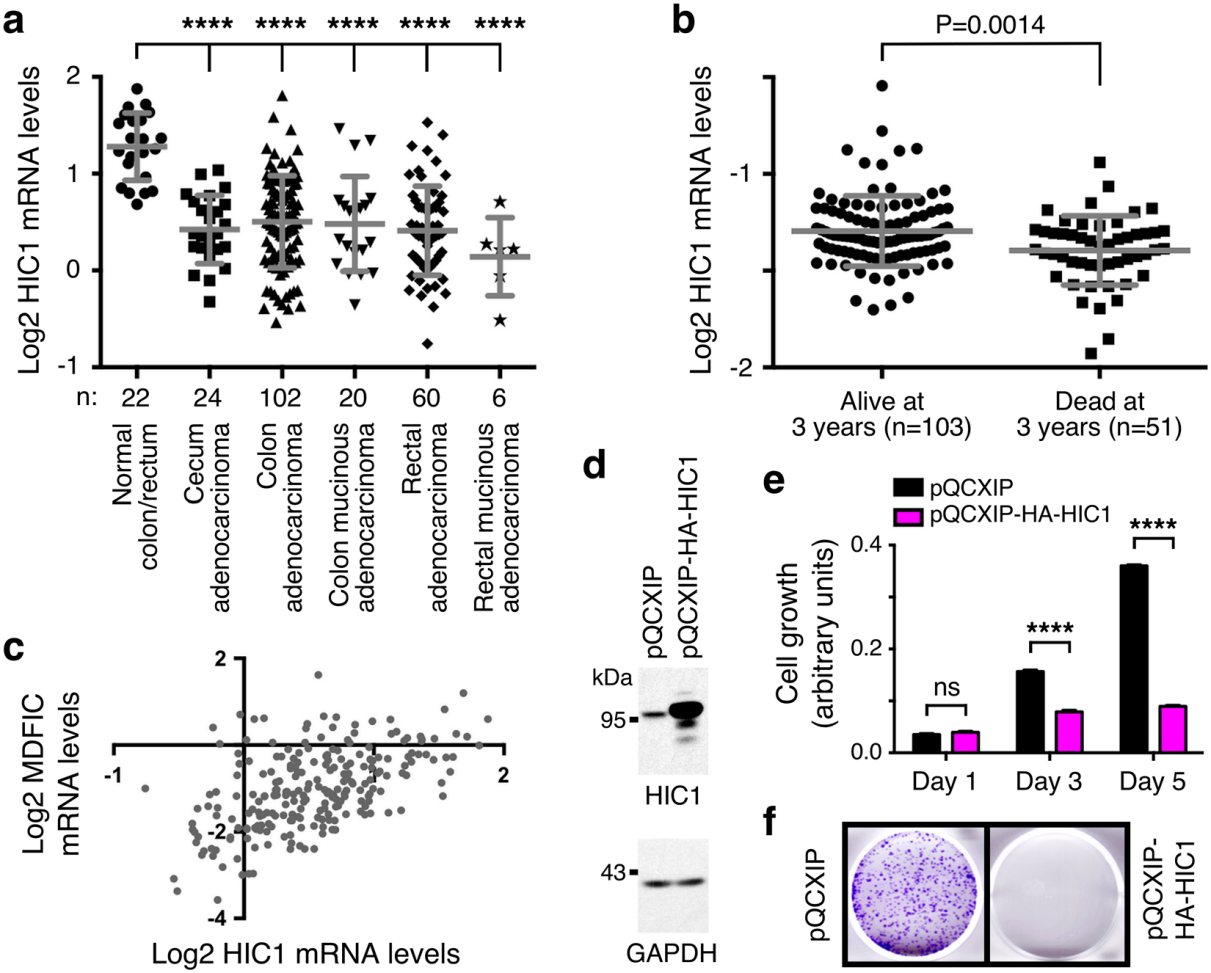
HIC1 in colorectal cancer. (**a**) *HIC1* mRNA levels in normal and diseased colorectal tissues. Data were derived from TCGA microarray experiments (reporter A_23_P129856). One-way ANOVA (Dunnett’s multiple comparisons test); ****P < 0.0001. (**b**) Association of *HIC1* mRNA levels (reporter 208461_at; data from Smith *et al*.^29^) with survival of colorectal cancer patients; unpaired, two-tailed t test. (**c**) Correlation between *HIC1* and *MDFIC* mRNA levels across all 234 samples in TCGA data shown in panel a of this figure and panel b of Fig. 2. Pearson correlation: r = 0.5376 (P < 0.0001). (**d**) Overexpression of HA-tagged HIC1 in HCT116 cells; pQCXIP represents the empty expression vector control. Shown are indicated Western blots. Uncropped Western blots are shown in Supplementary Fig. S9c. (**e**) Corresponding cell growth assay. Shown are means with standard deviations (n = 3). Two-way ANOVA (Tukey’s multiple comparisons test). ****P < 0.0001; ns, not significant. (**f**) Representative clonogenic assay.

We then focused on one gene strongly activated by MDFIC overexpression, namely *HIC1* that encodes for a DNA binding transcriptional repressor. The reasons were that the HIC1 protein is endowed with tumor suppressing activity^24^ and that *HIC1* (which stands for “hypermethylated in cancer 1”) is often epigenetically silenced in colorectal tumors^25–28^. Accordingly, we found in published microarray data^21,29^ that *HIC1* mRNA levels are downregulated in colorectal cancer, and low *HIC1* levels are associated with reduced survival and increased metastasis (Fig. 5a,b and Supplementary Fig. S10a–c). Of note, consistent with *HIC1* transcription being stimulated by MDFIC, *MDFIC* and *HIC1* levels strongly and positively correlated in colon (normal and cancerous) tissue (Fig. 5c and Supplementary Fig. S10d).

Next, we overexpressed HIC1 in HCT116 colorectal cancer cells (Fig. 5d and Supplementary Fig. S9c). Reduced cell growth (Fig. 5e) and clonogenic activity (Fig. 5f) were the consequences, mimicking the behavior of overexpressed MDFIC (see Fig. 3). This suggests that upregulation of *HIC1* could be an important means for MDFIC to suppress tumorigenesis. Consistently, downregulation of HIC1 blunted the ability of MDFIC to suppress HCT116 cell growth (Supplementary Fig. S11).

### MDFI and MDFIC expression in other cancers

**Figure 6:**
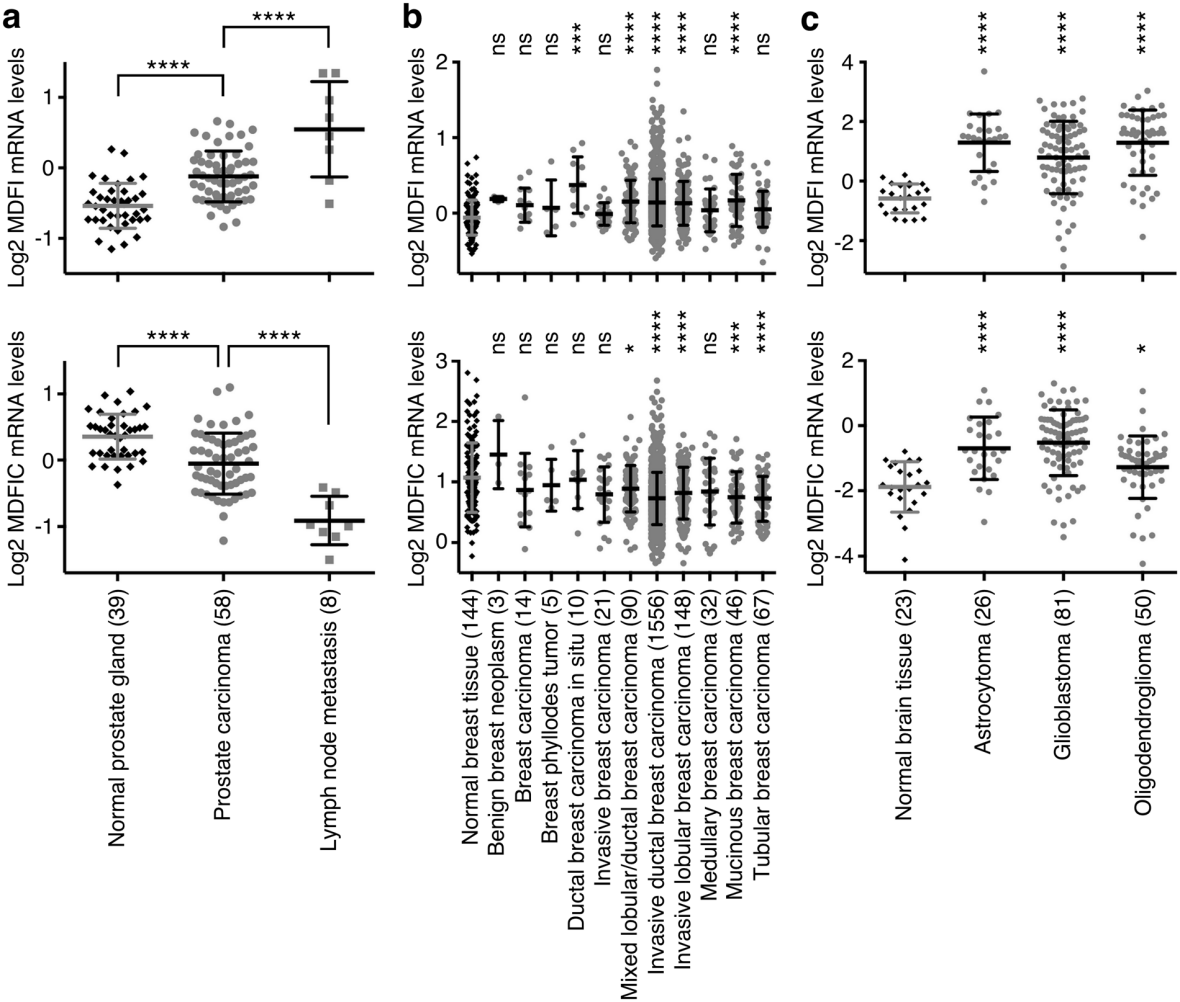
*MDFI* and *MDFIC* mRNA levels in various cancers. (**a**) *MDFI* or *MDFIC* mRNA levels in prostate cancer. Data were derived from Lapointe *et al*. (reporter IMAGE:33342 for *MDFI* and IMAGE:148810 for *MDFIC*)^30^. Number of specimens is indicated in parentheses. One-way ANOVA (Tukey’s multiple comparisons test). (**b**) Expression of *MDFI* and *MDFIC* in breast tumors; data from Curtis *et al*. (reporter ILMN_1782798 for *MDFI* and ILMN_1717366 for *MDFIC*)^31^. One-way ANOVA (Dunnett’s multiple comparisons test) was employed to assess differences with normal breast tissue. (**c**) Analogous in brain tumors; data from Sun et al. (reporter 205375_at for *MDFI* and 217599_s_at for *MDFIC*)^32^. *P < 0.05; ***P < 0.001; ****P < 0.0001; ns, not significant. In all panels, means with standard deviations are shown.

Finally, we wondered if *MDFI* and *MDFIC* may not only be involved in colorectal cancer, but also in neoplasms of other tissues. Using published microarray data^30–32^, we observed that, identical to colorectal tumors, *MDFI* and *MDFIC* exhibited up- and downregulation, respectively, in prostate, breast and ovarian cancer (Fig. 6a,b and Supplementary Fig. S12a). However, both *MDFI* and *MDFIC* were upregulated in brain, pancreatic and gastric tumors (Fig. 6c and Supplementary Fig. S12b,c). This implicates that MDFI may generally perform tumor promoting activities, whereas MDFIC might tissue-specifically inhibit or stimulate tumorigenesis. Furthermore, we observed again that *HIC1* and *MDFIC* mRNA levels were strongly correlated in breast, ovarian and gastric cancer and accordingly, like *MDFIC*, *HIC1* was downregulated in breast and ovarian tumors and upregulated in gastric tumors (Supplementary Fig. S13). This reinforces the notion that *HIC1* transcription may be regulated by MDFIC.

## Discussion

In this study, we provide the first evidence that MDFI and MDFIC are involved in colorectal cancer. Perplexingly, despite their high homology, they appear to act in opposite ways, namely MDFI as a tumor promoter and MDFIC as a repressor. This is based on the following evidence: First, we showed that *MDFI* is overexpressed, while *MDFIC* is downregulated in colorectal tumors, and high *MDFI* but low *MDFIC* levels are associated with more aggressive disease. Second, MDFI was capable of promoting HCT116 colon cancer cell growth, while MDFIC reduced it. Third, MDFI and MDFIC overexpression induced starkly different transcriptome changes. This included upregulation of the *HIC1* tumor suppressor gene upon MDFIC overexpression, while MDFI had no impact on *HIC1* expression.

Previous studies have shown that *MDFI* is hypermethylated in colorectal cancer, which would suggest reduced *MDFI* transcription^33,34^. This is in contrast to our bioinformatics results, showing upregulation of *MDFI* in colorectal tumors. However, methylation of only a few CpG sites in the *MDFI* gene promoter was previously examined, and it is unknown if these CpG sites are crucial for *MDFI* gene activity. On the other hand, *MDFIC* has been found to be deleted in myeloid disorders and thereby implied to be a candidate tumor suppressor^35^, which is in line with our data implicating a tumor suppressing function for MDFIC in the colon.

A second major finding presented in this manuscript is the physical interaction of both MDFI and MDFIC with JMJD1A, a histone demethylase known to perform oncogenic functions in colon cells^15–19^. Further, JMJD1A and MDFIC (or MDFI) regulated similar genes, and they could act in the same (e.g., *PDK4*) or opposite (e.g., *TGM2*) manner with regard to transcriptional activity. This implicates that the interaction of JMJD1A with MDFIC (or MDFI) has different effects on the activity of different promoters. However, this has the caveat that further studies are needed to substantiate that the observed effects of JMJD1A, MDFI or MDFIC on gene transcription were direct and not indirect. In this regard, our and published data^1,2,10,11^ showed that only a small fraction of MDFI and MDFIC resides in the cell nucleus, while the majority of these proteins is present in the cytoplasm. Hence, MDFI and MDFIC may plausibly perform many functions unrelated to transcriptional coregulation on the chromatin, yet may affect some transcriptional regulators in the cytoplasm. And indeed, previous studies have shown that MDFIC binds to and affects the glucocorticoid receptor in the cytoplasm^13^, or that MDFI and MDFIC interact with the cytoplasmic AXIN1 protein and in this manner modulate levels of the transcriptional cofactor β-catenin^4^. It is as well conceivable that MDFI and MDFIC regulate JMJD1A activity in the cytoplasm, since this histone demethylase is also present to a large extent in the cytoplasm of colon cancer cells^19^ where it may potentially demethylate cytoplasmic, non-histone proteins.

A third finding has been the identification of HIC1 as a seminal downstream effector of MDFIC. Indeed, HIC1 phenocopied MDFIC in suppressing HCT116 cell growth and clonogenic activity, suggesting that a MDFIC→HIC1 axis can restrain colorectal cancer development. Overexpression of MDFIC induced while JMJD1A downregulation decreased *HIC1* mRNA levels, suggesting that MDFIC and JMJD1A may cooperate to stimulate *HIC1* transcription. However, as neither JMJD1A nor MDFIC are endowed with DNA binding activity, it remains to be determined which *HIC1* regulating transcription factor(s) recruits JMJD1A and/or MDFIC to the *HIC1* gene locus. Consistent with our data showing that HIC1 overexpression suppressed HCT116 cell growth and clonogenic activity, depletion of *Hic1* in mice promoted both polyp formation in cooperation with loss of the tumor suppressor *Apc* and chemical carcinogenesis in the colon^36,37^. Mechanistically, HIC1 was shown to repress the *SIRT1* gene promoter, thereby preventing SIRT1-mediated deacetylation and inactivation of the tumor suppressor TP53^38^. However, another way how HIC1 can suppress tumorigenesis independent of TP53 is through averting chromosomal instability^39^. Therefore, it is tempting to hypothesize that MDFIC also regulates TP53 activity and genome integrity.

Our fourth, puzzling discovery is the fact that *MDFIC* expression in cancer can be either up- or downregulated depending on the organ. Accordingly, our bioinformatics results imply, but do not prove, that MDFIC context-dependently inhibits (breast, colon, ovary, prostate) or stimulates (brain, pancreas, stomach) tumorigenesis. Interestingly, we noted that MDFI and MDFIC can form homo- as well as heteromers, which is mediated by their conserved cysteine-rich C-terminal domains (Supplementary Fig. S14). Thus, one may speculate that high MDFI levels lead to sequestration of MDFIC into MDFI:MDFIC heteromers and thereby obstruct MDFIC function, while low MDFI levels would allow the formation of MDFIC homomers that only then could inhibit tumorigenesis in the breast, colon, ovary and prostate. Lastly, it is noteworthy that JMJD1A has been implicated in breast, gastric, ovarian and prostate cancer^40–45^, suggesting that the interaction of JMJD1A with MDFI/MDFIC is relevant beyond colorectal tumors.

## Methods

### Coimmunoprecipitation assay

Expression vectors for indicated proteins were transiently transfected into human 293 T embryonic kidney cells (ATCC CRL-3216) by the calcium phosphate coprecipitation method^46^. Approximately 40 h later, cells were lysed^47^ and immunoprecipitations performed as previously described^48^ with either anti-Flag M2 (Sigma-Aldrich F1804) or anti-HA 12CA5 (Santa Cruz Biotechnology sc-57592) mouse monoclonal antibodies. Immunoprecipitates as well as inputs were boiled in Laemmli sample buffer^49^ and subjected to polyacrylamide gel electrophoresis^50^. Separated proteins were transferred to polyvinylidene difluoride membranes^51^ and challenged with anti-Flag (Sigma-Aldrich F7425) or anti-Myc (Santa Cruz Biotechnology sc-789) rabbit polyclonal antibodies or anti-HA 12CA5 or anti-Myc 9E10 (Sigma-Aldrich M4439) mouse monoclonal antibodies^52^. This was followed by incubation with horseradish peroxidase-coupled secondary antibodies^53^ and signal detection through enhanced chemiluminescence^54^.

### *In vitro* protein binding assay

Fusions of MDFI or MDFIC with glutathione *S*-transferase (GST) were expressed in *Escherichia coli* BL21 Codon-Plus (Stratagene)^55^ and affinity purified with the help of glutathione agarose^56^. Flag- and 6His-tagged JMJD1A was expressed with the help of baculovirus in Sf9 insect cells (Bac-to-Bac system, Invitrogen) and purified on Ni^2+^-NTA agarose (Qiagen)^57^. After binding of GST fusion proteins to glutathione agarose, these beads were challenged with purified JMJD1A^58^ and bound JMJD1A subsequently revealed by anti-Flag Western blotting^59^.

### Generation of virally transduced cells

Retro-/lentivirus was produced in 293 T cells according to standard procedures^60^. Then, human HCT116 colorectal cancer cells (ATCC CCL-247) were thrice infected within 24 h^61^, split 24 h later and selected for two days with 200 µg/ml hygromycin B or 1 µg/ml puromycin^62^. RNA interference targeted the following sequences within the human *JMJD1A* open reading frame: GCAGGUGUCAAUAGUGAUA (shRNA #1), GUAGACCUAGUUAAUUGUA (shRNA #2) and CUGCAAAGGACACGGAGAA (shRNA #3). Doxycycline-inducible HCT116 cells were created by viral transduction as described^63^.

### Cell growth and clonogenic assay

2400 virally transduced HCT116 cells were seeded into 96-wells after two days of selection with hygromycin B or puromycin and their growth assessed over the next 1–5 days as described^64^. Likewise, 2400 virally transduced HCT116 cells were seeded into 6-wells and clonogenic activity revealed approximately 10 days thereafter by staining with crystal violet^65^.

### RNA sequencing and validation

Total RNA was isolated as described^19^ and then subjected to RNA sequencing at Novogene (https://en.novogene.com). Differential expression analysis of two conditions was performed using version 3.16.5 of the edgeR software package^66^ and the P values were adjusted using the Benjamini & Hochberg method. Validation of differentially expressed genes was done with RT-PCR^67^ and visualization of amplified DNA fragments through ethidium-bromide staining^68^ after agarose gel electrophoresis^69^. Respective primers are listed in *Supplementary Information*.

### Statistics

Statistical tests that were used are described in the figure legends. Where applicable, means with standard deviations are presented in the figures. All calculations were done with GraphPad Prism 6 for Mac OS X. Statistical significance was assumed for P values less than 0.05.

## Supplementary information

Supplementary information.

## Data Availability

Data, detailed protocols and DNA constructs will be made available upon reasonable request. RNA sequencing data have been deposited in the NCBI BioProject database under accession number PRJNA551463 and can be freely downloaded from the NCBI Sequence Read Archive (accession numbers SRX6406581, SRX6406582, SRX6406583, SRX6406584, SRX6406585, SRX6406586, SRX6406587, SRX6406588 and SRX6406589).
