# Supplementary material for: Opposite Roles of the JMJD1A Interaction Partners MDFI and MDFIC in Colorectal Cancer
Source: Sci Rep. 2020 May 26;10:8710. doi: 10.1038/s41598-020-65536-6 (PMC7250871; doi:10.1038/s41598-020-65536-6)
Supplement: Supplementary file 1 — Supplementary information. [file 41598_2020_65536_MOESM1_ESM.pdf]

SUPPLEMENTARTY INFORMATION

Opposite Roles of the JMJD1A Interaction Partners MDFI and MDFIC in Colorectal Cancer

Yuan Sui, Xiaomeng Li, Sangphil Oh, Bin Zhang,

Willard M. Freeman, Sook Shin, Ralf Janknecht

Contents:

(I) Supplementary Materials

- List of Used RT-PCR Primers
- List of Used Primary Antibodies

(II) Supplementary Figures and Legends

(III) Supplementary References

(I) Supplementary Materials

List of Used RT-PCR Primers

Gene	Forward primer (5’ → 3’)	Reverse primer (5’ → 3’)	bp
PDK4	GGTCCAAGATGCCTTTGAGTG	TGGCAAGCCGTAACCAAAAC	397
SERPINE1	GGGCCATGGAACAAGGATGA	GGGCGTGGTGAACTCAGTATAG	399
TGM2	ACTTTGAGGGCCGCAACTAC	AGGTACACAGCATCCGCTGG	319
CTGF	CGTGTGCACCGCCAAAGATG	ACCAGGCAGTTGGCTCTAATC	324
RCAN2	TGACTGTGTGACGTTCCAGC	GCTCATACTTCTCTCCTGGTCC	329
ZEB2	GGAGGAAAAACGTGGTGAACT	TAATTGCGGTCTGGATCGTGG	330
HIC1	CACACAGGTGCAGAGGCTTG	AGGACTATGCCAGAAGCGATG	316
GAPDH	GAGCCACATCGCTCAGACACC	TGACAAGCTTCCCGTTCTCAGC	226
MDFI	CTGGAGGTAGTAACAGGATCCACTC	CGATGTTGCACAGCGTCAGGAACTC	445
MDFIC	GGAAATCCTTCGGATGGTGAACTC	CAAGCAAGCCAGGATACAGTGGACAC	339

List of Used Primary Antibodies

Flag M2 mouse monoclonal antibodies	Sigma-Aldrich F1804
Flag rabbit polyclonal antibodies	Sigma-Aldrich F7425
GAPDH goat polyclonal antibodies	Genscript A00191-40
H3K27me$_1$ rabbit polyclonal antibodies	Upstate Biotechnology 07-448
HA 12CA5 mouse monoclonal antibodies	Santa Cruz Biotechnology sc-57592
HIC1 rabbit polyclonal antibodies	Sigma-Aldrich AV32623
JMJD1A rabbit polyclonal antibodies	Novus Biologicals NB100-77282
Lamin B goat polyclonal antibodies	Santa Cruz Biotechnology sc-6216
Myc 9E10 mouse monoclonal antibodies	Sigma-Aldrich M4439
Myc rabbit polyclonal antibodies	Santa Cruz Biotechnology sc-789

(II) Supplementary Figures and Legends

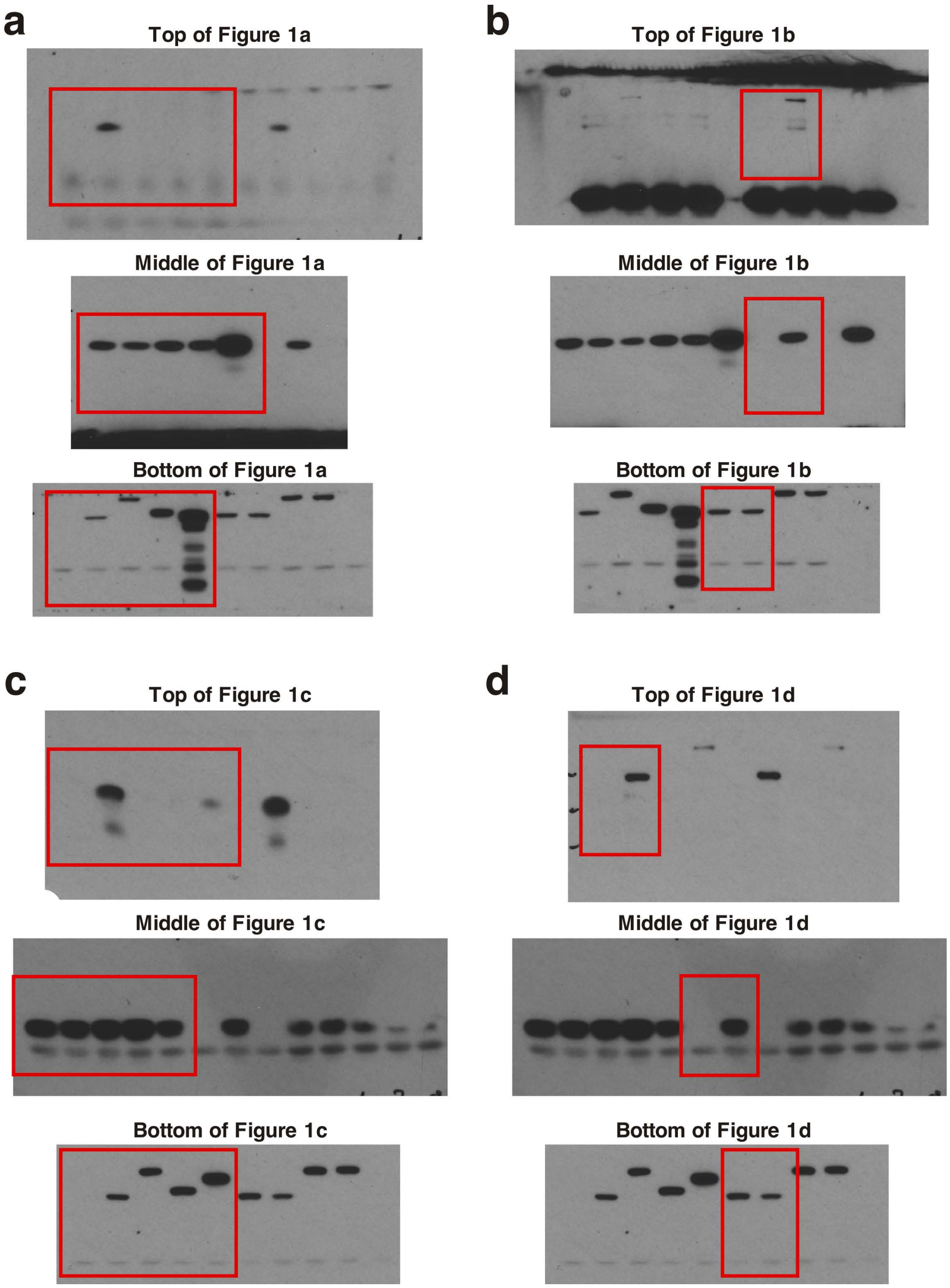

Supplementary Figure S1. Uncropped images for which boxed areas are shown in the published panels a-d of Figure 1. (**a**) Corresponding to Figure 1a. (**b**) Corresponding to Figure 1b. (**c**) Corresponding to Figure 1c. (**d**) Corresponding to Figure 1d.

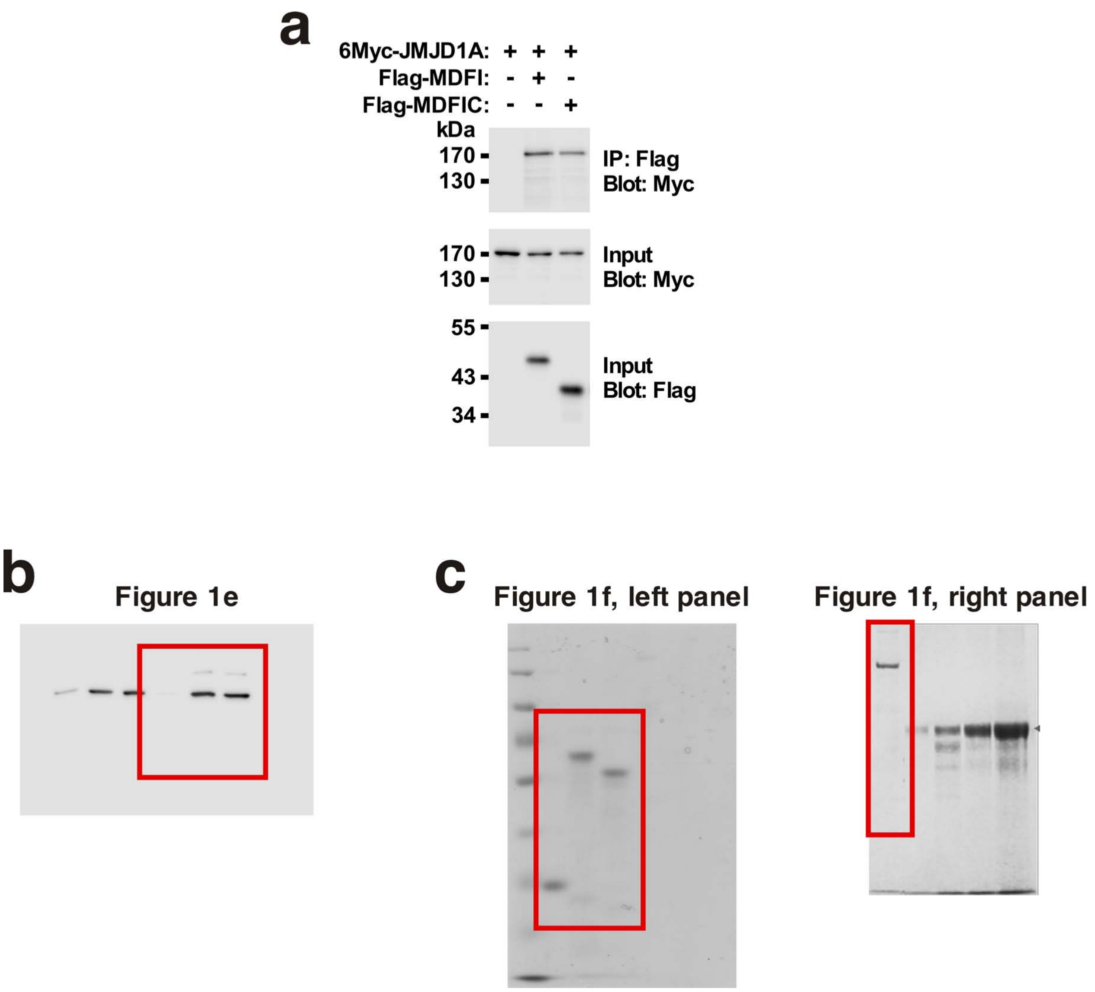

Supplementary Figure S2. (**a**) Coimmunoprecipitation of 6Myc-tagged JMJD1A with either Flag-tagged MDFI or MDFIC in 293T cells. Top shows immunoprecipitation (IP) with anti-Flag antibodies, while the bottom two panels show input levels of Myc- and Flag-tagged proteins. (**b**) Uncropped image corresponding to Figure 1e; boxed area is shown in Figure 1e. (**c**) Analogous, uncropped images corresponding to Figure 1f.

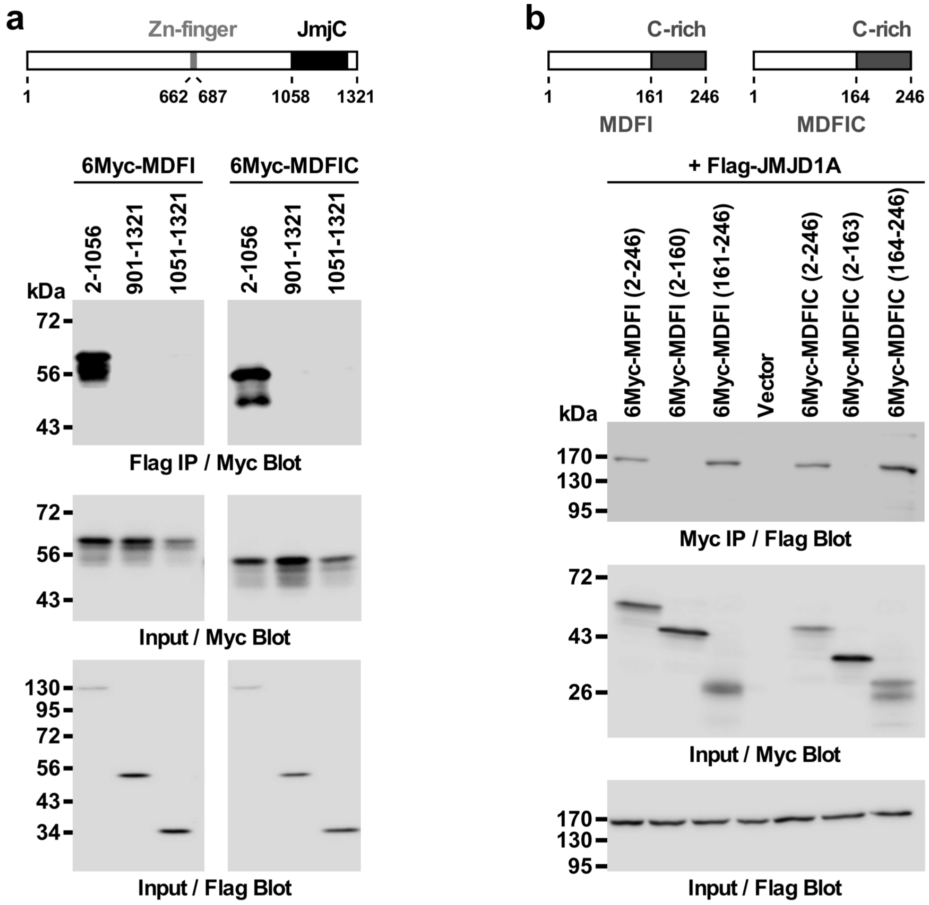

c

```
MDFI   MYQVSGQRPSGCDAPYGAPSAAPG--PAQTLSLLPGLEVVTGSTHPAEAAPEEGSLEEAATPMPQGNGPG  68
MDFIC  MSGAGEALAPGPVGPQRVAEAGGGQLGSTAQGKCDKDNTEKDITQATNSHFTHGEMQDQSIWGNPSDGEL  70
       *         *   *     *  *                   *         *             *

MDFI   I-PQGLDSTDLDVPTEAVTCQPQGNPLGCTPLLPNDSG--HPSELGGTRRAGNGALGGPKAHRKLQTHPS  135
MDFIC  IRTQPQRLPQLQTSAQVPSGEEIGKIKNGHTGLSNGNGIHHGAKHGSADNRKLSAPVSQKMHRKIQSSLS  140
       *  *      *            *        * *  *  *    *        *    * *** *   *

MDFI   LASQGSKKSKSSSKSTTSQIPLQAQEDCCVHCILSCLFCEFLTLCNIVLDCATCGSCSSEDSCLCCCCCG  205
MDFIC  VNSDISKKSKVN--AVFSQKTGSSPEDCCVHCILACLFCEFLTLCNIVLGQASCGICTSE---ACCCCCG  205
         *  *****       **      **$$**$** $**$*****$****  * $* $ **    $$$$$*

MDFI   SGECADCDLPCDLDCGILDACCESADCLEICMECCGLCFSS                              246
MDFIC  DEMGDDCNCPCDMDCGIMDACCESSDCLEICMECCGICFPS                              246
            *$  *$* *$** **$$** *$***$**$$* $* *
```

Supplementary Figure S3. Mapping of interaction domains. (**a**) Coimmunoprecipitation of 6Myc-tagged MDFI or MDFIC with indicated Flag-tagged JMJD1A amino acids. The top shows a sketch of the human JMJD1A protein with its Zn-finger and the catalytic center, the Jumonji C (JmjC) domain. (**b**) Coimmunoprecipitation of Flag-JMJD1A with indicated 6Myc-MDFI and MDFIC amino acids. Schematic structures of MDFI and MDFIC are outlined at the top; C-rich, cysteine-rich C-terminal domain. (**c**) Amino acid alignment of human MDFI (NCBI NP_001287735.1) and MDFIC (NCBI NP_001159817.1). The highly homologous C-terminal domains (amino acids 161-246 and 164-246 of MDFI and MDFIC, respectively) are highlighted in grey. Conserved cysteines are marked by "$", and other conserved amino acids by asterisks.

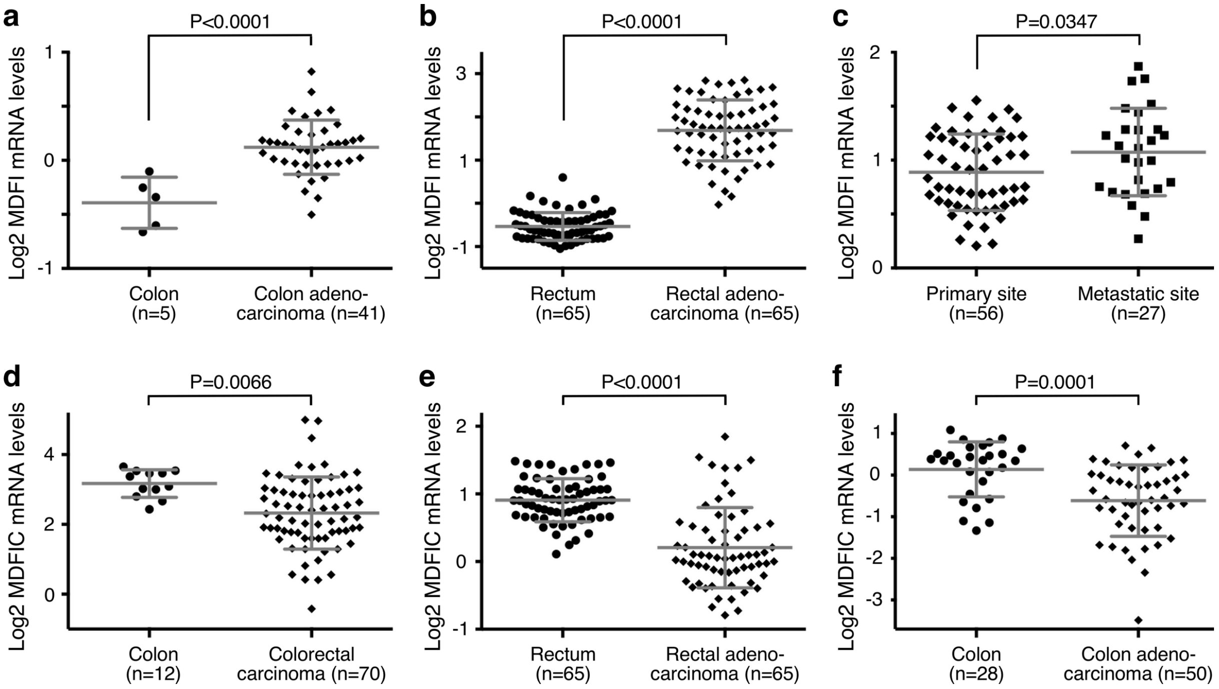

Supplementary Figure S4. *MDFI* mRNA upregulation in colorectal tumors compared to healthy colon or rectum; (**a**) data from Kaiser *et al* (1) with probe 205375_at or (**b**) from Gaedcke *et al* (2) with probe A_23_P42168. (**c**) Increased expression of *MDFI* at metastatic compared to primary sites in colorectal carcinomas. Data from Tsuji *et al* (3) with probe 205375_at. (**d-f**) Downregulation of *MDFIC* in colon cancer detected in microarray data from Hong *et al* (4) with probe 211675_s_at, from Gaedcke *et al* (2) with probe A_23_P327022, and from Ki *et al* (5) with probe N79548, respectively. Shown are means with standard deviations. Unpaired, two-tailed t test was utilized to assess statistical significance.

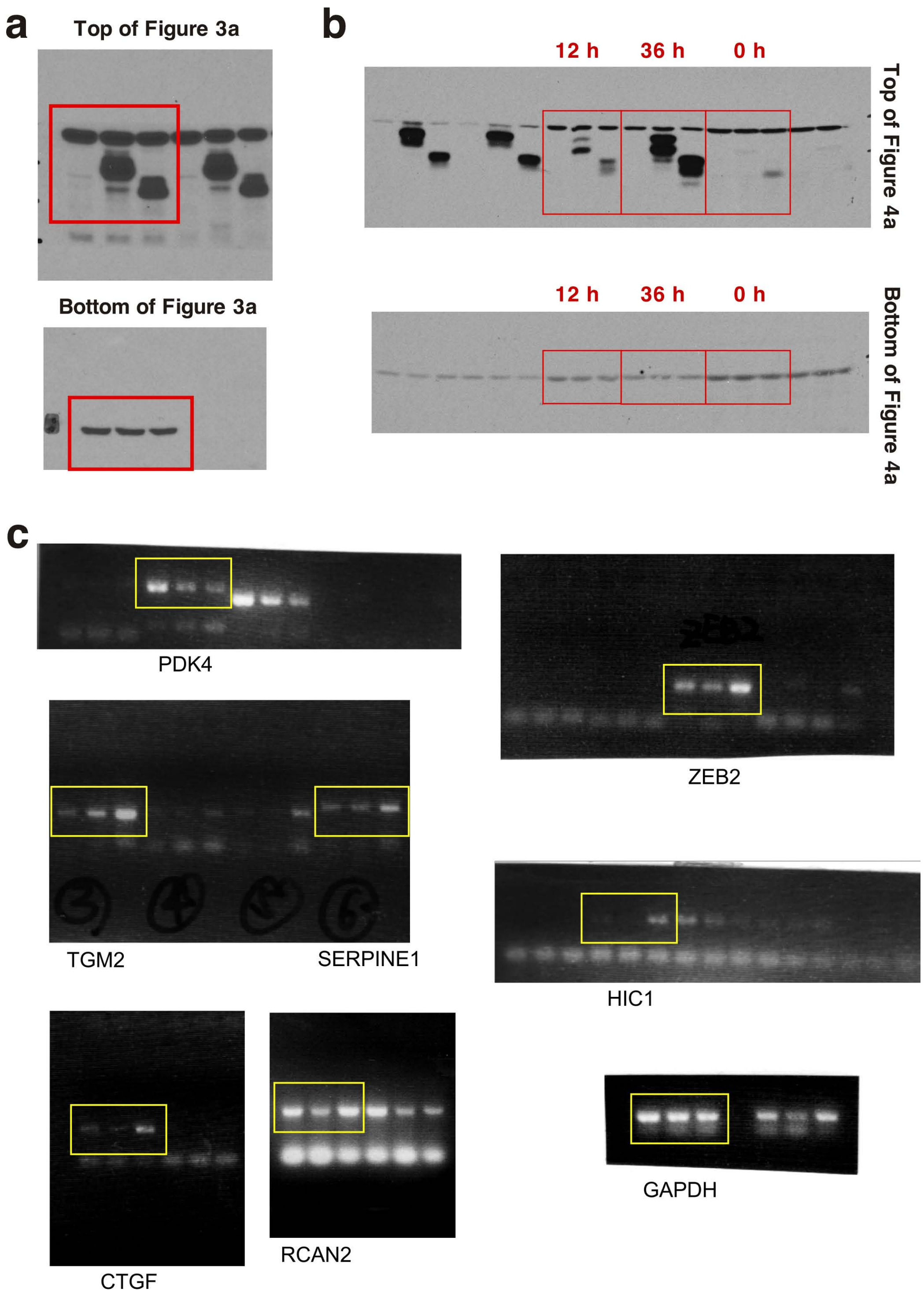

Supplementary Figure S5. Uncropped images for which boxed areas are shown in the indicated published figures. (**a**) Corresponding to Figure 3a. (**b**) Corresponding to Figure 4a. (**c**) Corresponding to Figure 4c.

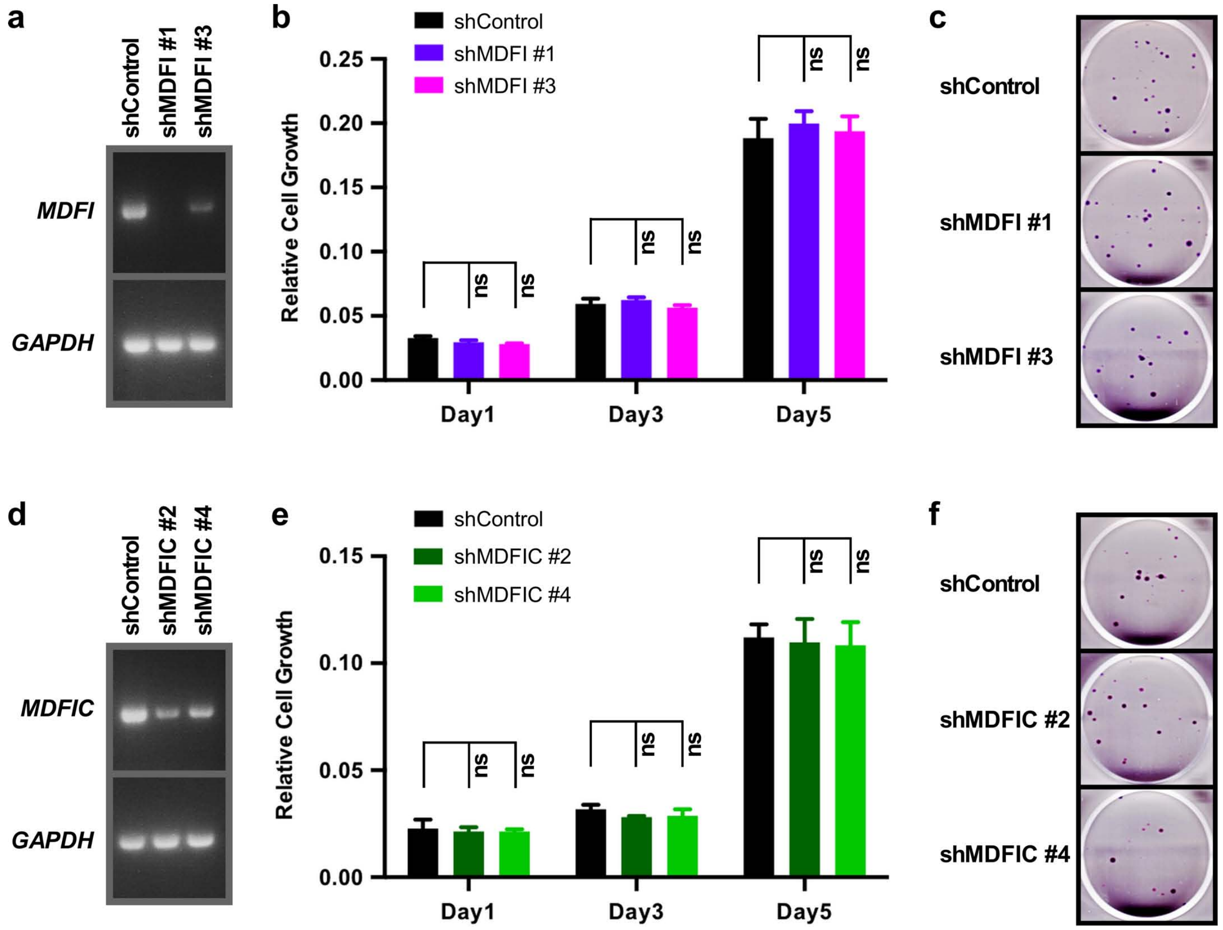

Supplementary Figure S6. Downregulation of MDFI or MDFIC in HCT116 cells. (**a**) RT-PCR demonstrating downregulation of *MDFI* mRNA upon expression of MDFI shRNA #1 (targeting GCUGAGGACCUGACAGGACAA) or #3 (targeting UGUCACACAAGGCUUGAGAAG). (**b**) Growth assays. Two-way ANOVA (Tukey's multiple comparison tests); shown are means with standard deviations (n=3). ns, not significant. (**c**) Representative clonogenic assay. (**d-f**) Analogous upon downregulation of MDFIC with shRNA #2 (targeting AGACUCCAUCUCUAAAUAAAU) or shRNA #4 (targeting UCAGUCCAGCUUGUCUGUAAA).

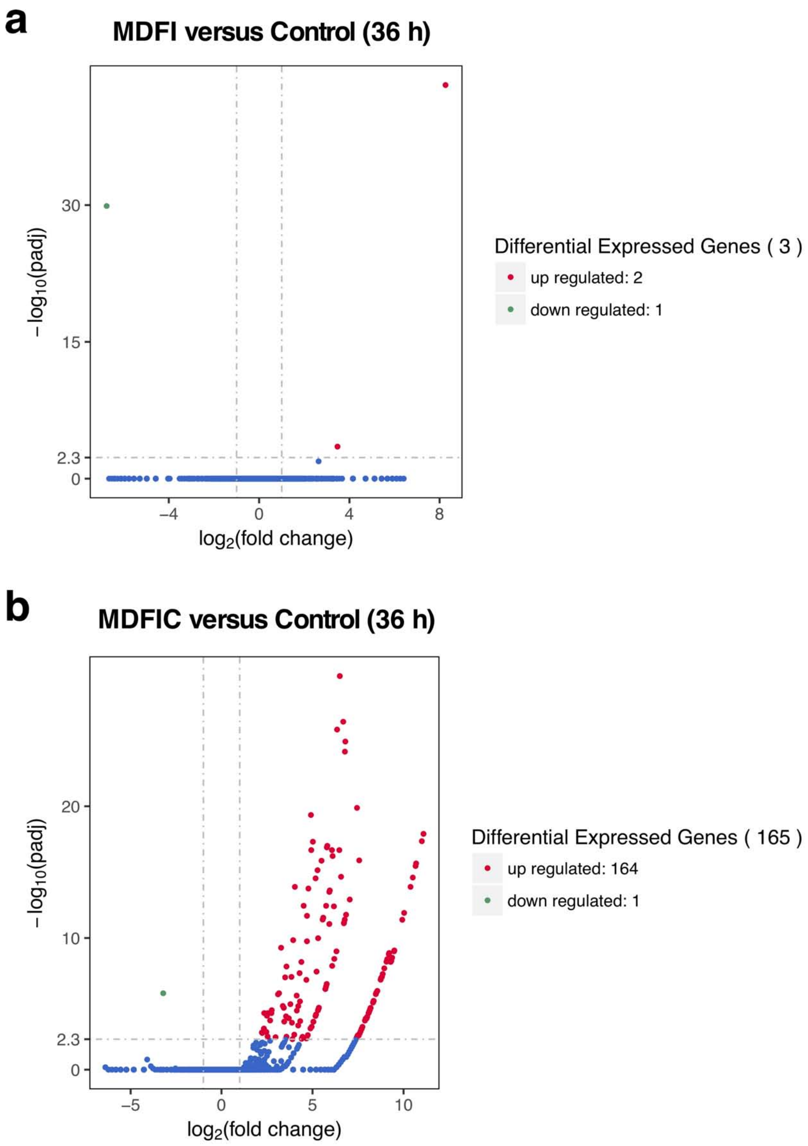

Supplementary Figure S7. Volcano diagram of differential gene expression revealed in RNA sequencing experiments. The threshold was set at an adjusted P value of less than 0.005 and $|\log_2(\text{fold change})| > 1$. (**a**) MDFI- or (**b**) MDFIC-overexpressing cells versus control HCT116 cells after 36 h of doxycycline induction.

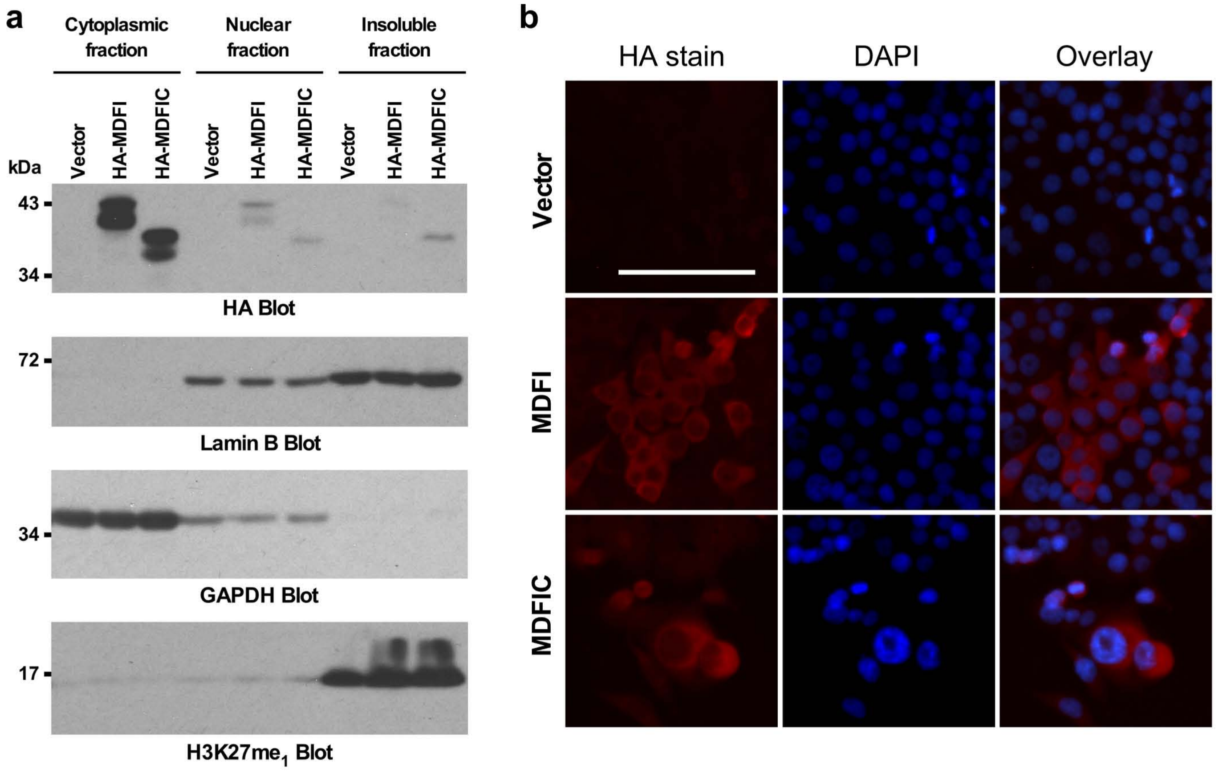

Supplementary Figure S8. MDFI and MDFIC intracellular localization in HCT116 colorectal cancer cells. (**a**) Cells transduced with retrovirus expressing HA-tagged MDFI or MDFIC were fractionated with the NE-PER nuclear and cytoplasmic extraction kit (Pierce Biotechnology 78833). Shown are indicated Western blots. (**b**) Corresponding cell staining with anti-HA antibodies. Nuclei were additionally stained with DAPI. Scale bar = 100 μm.

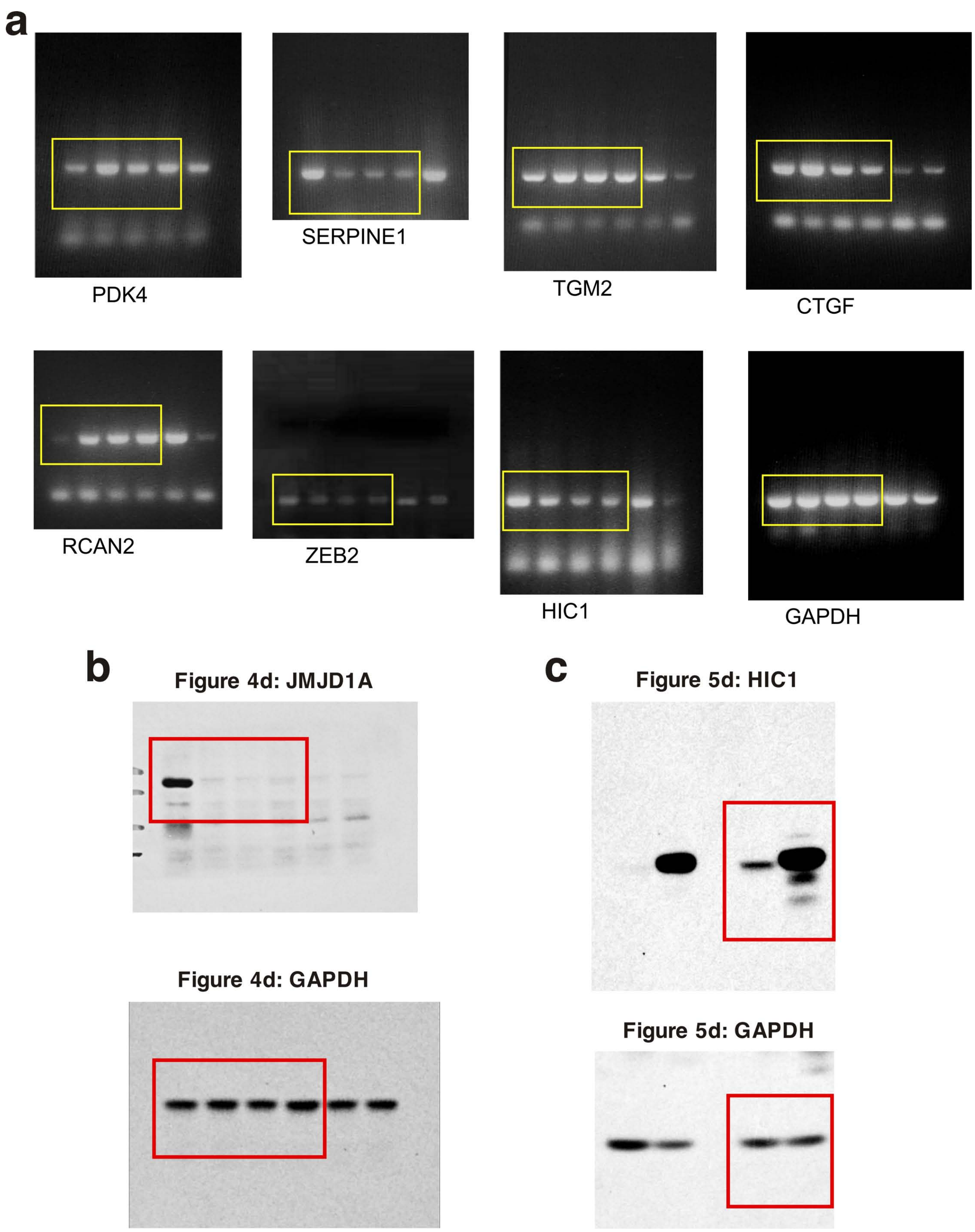

Supplementary Figure S9. Uncropped images for which boxed areas are shown in the indicated published figures. (**a**) Corresponding to Figure 4d (agarose gels). (**b**) Corresponding to Figure 4d (Western blots). (**c**) Corresponding to Figure 5d (Western blots).

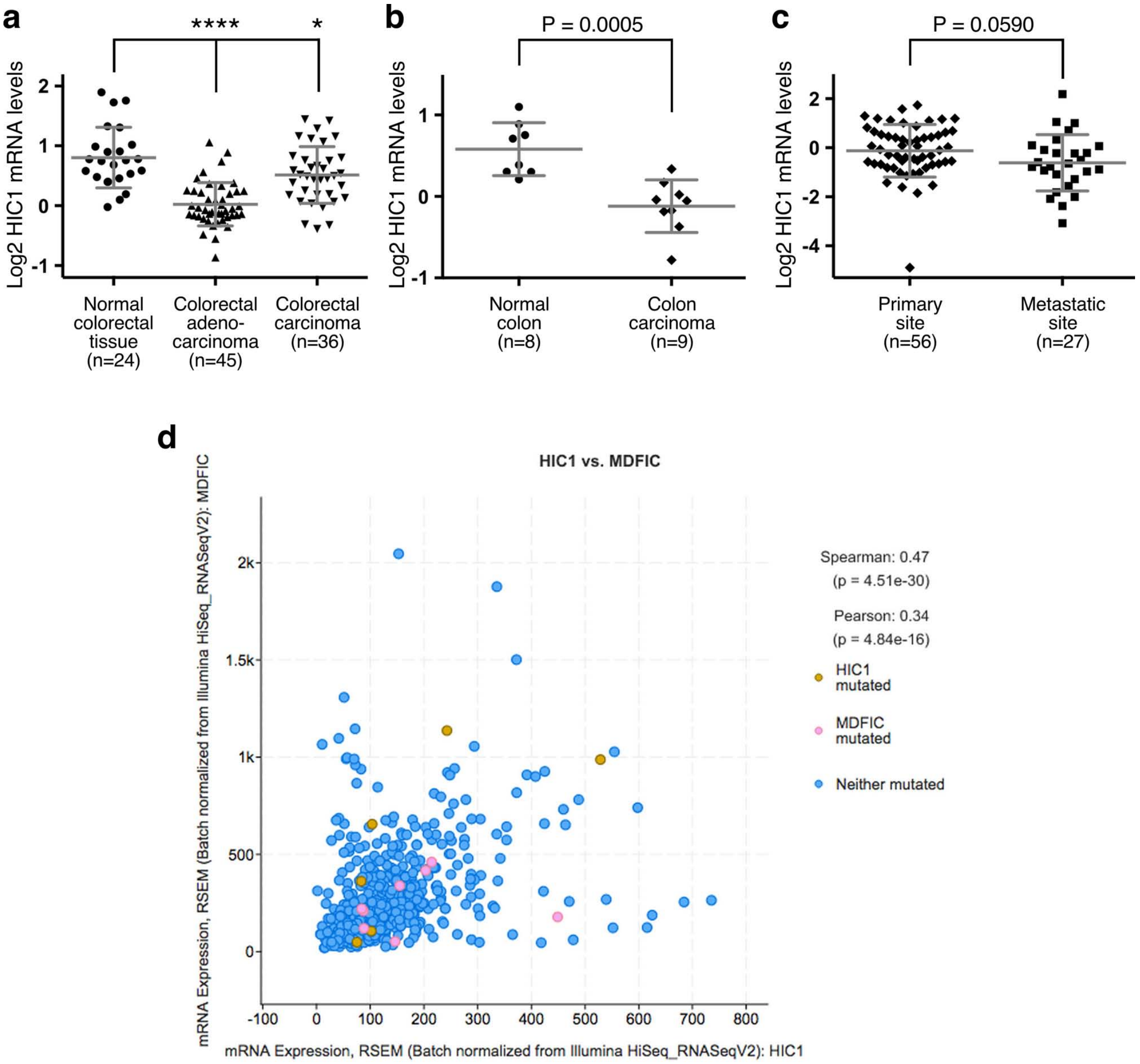

Supplementary Figure S10. *HIC1* mRNA levels in colorectal tumors. (**a**) Data from Skrzypczak *et al* (6) with probe 230218_at or (**b**) from Zou *et al* (7) with probe IMAGE:856887 or (**c**) from Tsuji *et al* (3) with probe 230218_at. Shown are means with standard deviations. One-way ANOVA (Dunnett's multiple comparisons test) was utilized for panel a, and unpaired, two-tailed t test for panels b and c. *, $P<0.05$; ****, $P<0.0001$. (**d**) Correlation of *HIC1* and *MDFIC* mRNA levels in TCGA PanCancer Atlas for colorectal adenocarcinomas (n=524); RNA sequencing data were analyzed with cBioPortal.

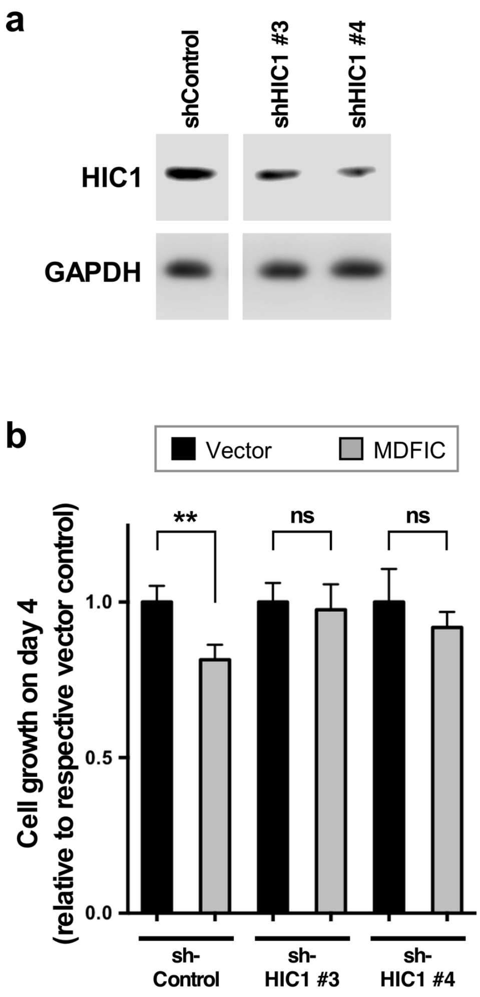

Supplementary Figure S11. Downregulation of HIC1 blunts MDFIC activity in HCT116 cells. (**a**) Western blots showing downregulation of HIC1 with two different shRNAs, which targeted the sequence GGAAGAGUAGAGUUAAGAUGA (#3) or GCUAUAGUGGCACAGAUAUCU (#4). (**b**) Corresponding cell growth assays. For each shRNA group, growth on day 4 was normalized to the respective vector control (which was set to 1). Shown are means with standard deviations (n=4). One-way ANOVA (Sidak's multiple comparisons test); **, P<0.01; ns, not significant. Please note that the degree of MDFIC overexpression is expected to be less than in Figure 3 due to only one time instead of three times infecting cells with respective virus, resulting in less growth inhibition.

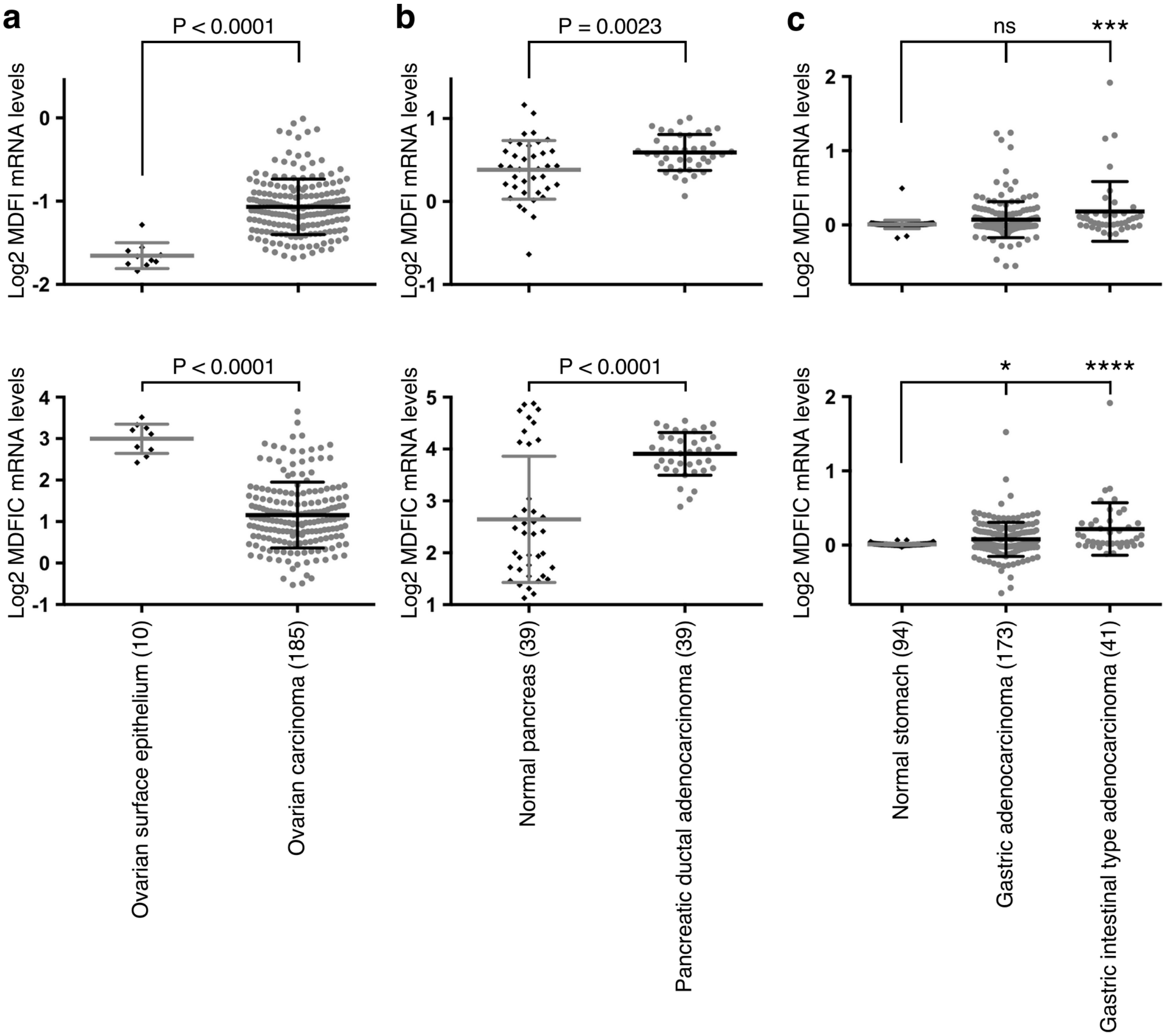

Supplementary Figure S12. *MDFI* and *MDFIC* mRNA levels in ovarian, pancreatic and gastric cancer. Shown are means with standard deviations. (**a**) *MDFI* or *MDFIC* mRNA levels in ovarian carcinoma. Data were derived from Bonome *et al* (reporter 205375_at for *MDFI* and 211675_s_at for *MDFIC*) (8). Number of specimens is indicated in parentheses. Unpaired, two-tailed t test. (**b**) Likewise, expression of *MDFI* and *MDFIC* in pancreatic ductal adenocarcinoma; data from Badea *et al* (reporter 205375_at for *MDFI* and 211675_s_at for *MDFIC*) (9). Unpaired, two-tailed t test. (**c**) Analogous in gastric tumors; data from TCGA (reporter 06-041722066 for *MDFI* and 07-114397972 for *MDFIC*). One-way ANOVA (Dunnett's multiple comparisons test) was employed to assess differences with normal stomach tissue. *, $P<0.05$; ***, $P<0.001$; ****, $P<0.0001$; ns, not significant.

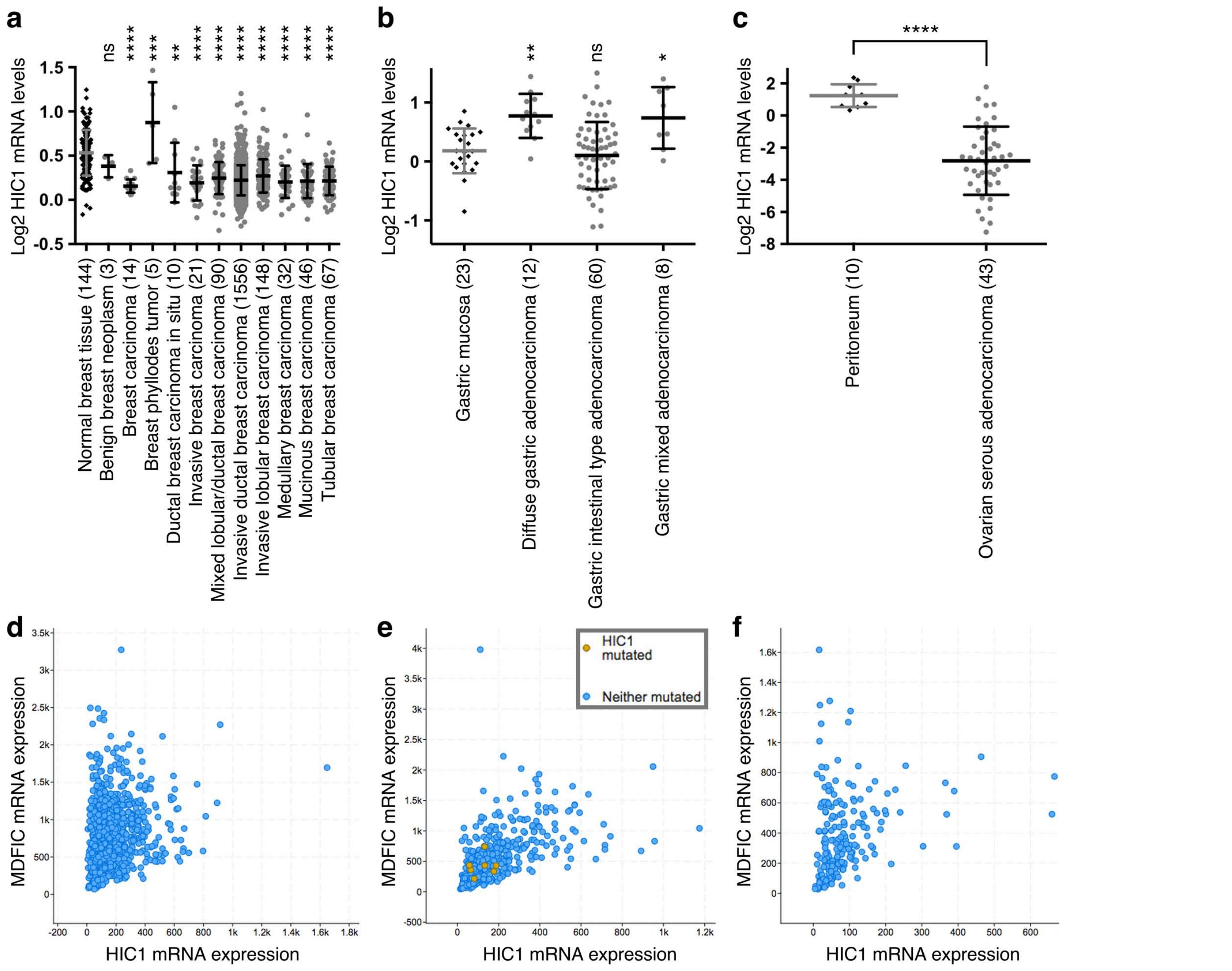

Supplementary Figure S13. *HIC1* mRNA levels in breast (**a**), gastric (**b**) and ovarian cancer (**c**). Shown are means with standard deviations. Number of samples is indicated in parentheses. Data were derived from Curtis *et al* (reporter ILMN_1738825) (10), Chen *et al* (reporter IMAGE:856887) (11) and Yoshihara *et al* (reporter A_23_P129856) (12), respectively. One-way ANOVA (Dunnett's multiple comparisons test) was applied for panels a and b, and unpaired, two-tailed t test for panel c. *, P<0.05; **, P<0.01; ***, P<0.001; ****, P<0.0001; ns, not significant. (**d-f**) Correlation of *HIC1* and *MDFIC* mRNA expression in TCGA PanCancer Atlas for breast invasive carcinoma (n=996; Pearson r=0.21; P=1.9e-11), stomach adenocarcinoma (n=407; Pearson r=0.53; P=7.16e-31) and ovarian serous cystadenocarcinoma (n=201; Pearson r=0.25; P=3.799e-4), respectively. RNA sequencing data (RSEM, batch normalized from Illumina HiSeq_RNASeqV2) were analyzed with cBioPortal.

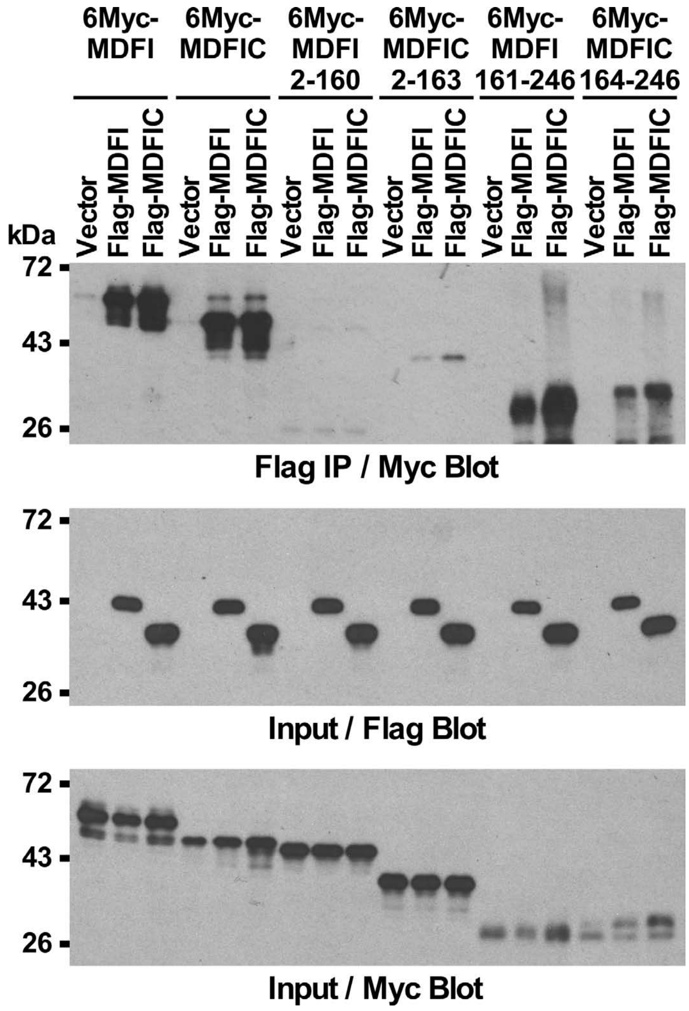

Supplementary Figure S14. Oligomerization of MDFI and MDFIC. Indicated 6Myc- or Flag-tagged proteins were coexpressed in 293T cells and anti-Flag immunoprecipitations (IP) performed. Coprecipitated proteins were revealed by anti-Myc Western blotting. The bottom two panels show input levels of the respective Flag- and 6Myc-tagged proteins. The left three lanes show that Myc-tagged MDFI coprecipitated with Flag-MDFI and Flag-MDFIC, but not with the vector control. Similarly, Myc-tagged MDFIC coprecipitated with Flag-MDFI and Flag-MDFIC (lanes 4-6). Neither MDFI amino acids 2-160 nor MDFIC amino acids 2-163 were capable of robustly forming complexes with full-length MDFI or MDFIC (lanes 7-12), but the C-termini did (lanes 13-18), indicatating that the formation of homo- and heteromeric complexes is primarily mediated by the conserved C-termini of MDFI and MDFIC.

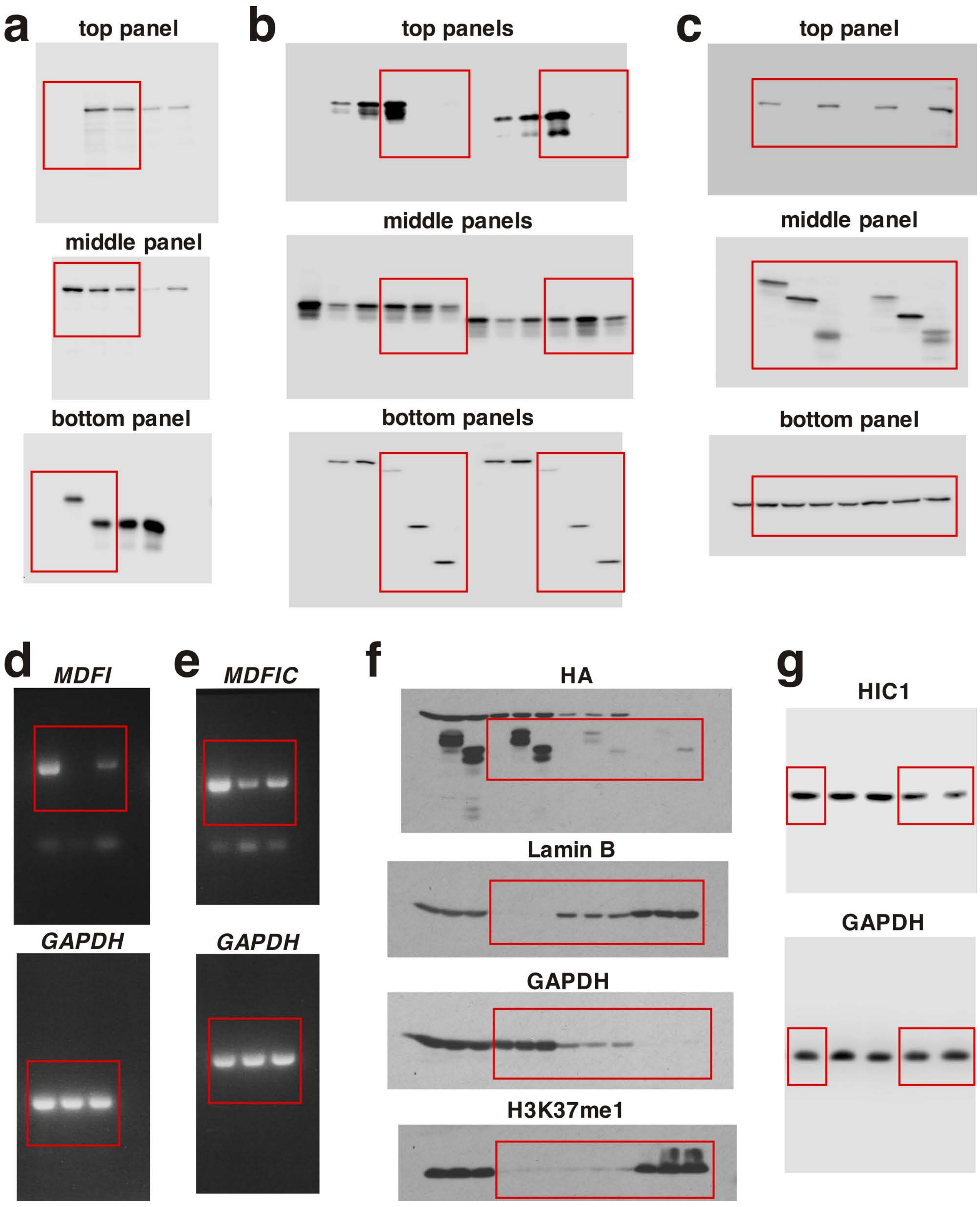

Supplementary Figure S15. Uncropped images corresponding to Supplementary Figures S2a (**a**), S3a (**b**), S3b (**c**), S6a (**d**), S6d (**e**), S8a (**f**) and S11a (**g**).

(III) Supplementary References

1. Kaiser, S., Park, Y. K., Franklin, J. L., Halberg, R. B., Yu, M., Jessen, W. J., Freudenberg, J., Chen, X., Haigis, K., Jegga, A. G., Kong, S., Sakthivel, B., Xu, H., Reichling, T., Azhar, M., Boivin, G. P., Roberts, R. B., Bissahoyo, A. C., Gonzales, F., Bloom, G. C., Eschrich, S., Carter, S. L., Aronow, J. E., Kleimeyer, J., Kleimeyer, M., Ramaswamy, V., Settle, S. H., Boone, B., Levy, S., Graff, J. M., Doetschman, T., Groden, J., Dove, W. F., Threadgill, D. W., Yeatman, T. J., Coffey, R. J., Jr., and Aronow, B. J. (2007) Transcriptional recapitulation and subversion of embryonic colon development by mouse colon tumor models and human colon cancer. *Genome Biol.* 8, R131
2. Gaedcke, J., Grade, M., Jung, K., Camps, J., Jo, P., Emons, G., Gehoff, A., Sax, U., Schirmer, M., Becker, H., Beissbarth, T., Ried, T., and Ghadimi, B. M. (2010) Mutated KRAS results in overexpression of DUSP4, a MAP-kinase phosphatase, and SMYD3, a histone methyltransferase, in rectal carcinomas. *Genes Chromosomes Cancer* 49, 1024-1034
3. Tsuji, S., Midorikawa, Y., Takahashi, T., Yagi, K., Takayama, T., Yoshida, K., Sugiyama, Y., and Aburatani, H. (2012) Potential responders to FOLFOX therapy for colorectal cancer by Random Forests analysis. *Br. J. Cancer* 106, 126-132
4. Hong, Y., Downey, T., Eu, K. W., Koh, P. K., and Cheah, P. Y. (2010) A 'metastasis-prone' signature for early-stage mismatch-repair proficient sporadic colorectal cancer patients and its implications for possible therapeutics. *Clin. Exp. Metastasis* 27, 83-90
5. Ki, D. H., Jeung, H. C., Park, C. H., Kang, S. H., Lee, G. Y., Lee, W. S., Kim, N. K., Chung, H. C., and Rha, S. Y. (2007) Whole genome analysis for liver metastasis gene signatures in colorectal cancer. *Int. J. Cancer* 121, 2005-2012
6. Skrzypczak, M., Goryca, K., Rubel, T., Paziewska, A., Mikula, M., Jarosz, D., Pachlewski, J., Oledzki, J., and Ostrowski, J. (2010) Modeling oncogenic signaling in colon tumors by multidirectional analyses of microarray data directed for maximization of analytical reliability. *PLoS One* 5, e13091
7. Zou, T. T., Selaru, F. M., Xu, Y., Shustova, V., Yin, J., Mori, Y., Shibata, D., Sato, F., Wang, S., Olaru, A., Deacu, E., Liu, T. C., Abraham, J. M., and Meltzer, S. J. (2002) Application of cDNA microarrays to generate a molecular taxonomy capable of distinguishing between colon cancer and normal colon. *Oncogene* 21, 4855-4862
8. Bonome, T., Levine, D. A., Shih, J., Randonovich, M., Pise-Masison, C. A., Bogomolniy, F., Ozbun, L., Brady, J., Barrett, J. C., Boyd, J., and Birrer, M. J. (2008) A gene signature predicting for survival in suboptimally debulked patients with ovarian cancer. *Cancer Res.* 68, 5478-5486
9. Badea, L., Herlea, V., Dima, S. O., Dumitrascu, T., and Popescu, I. (2008) Combined gene expression analysis of whole-tissue and microdissected pancreatic ductal adenocarcinoma identifies genes specifically overexpressed in tumor epithelia. *Hepatogastroenterology* 55, 2016-2027
10. Curtis, C., Shah, S. P., Chin, S. F., Turashvili, G., Rueda, O. M., Dunning, M. J., Speed, D., Lynch, A. G., Samarajiwa, S., Yuan, Y., Graf, S., Ha, G., Haffari, G., Bashashati, A., Russell, R., McKinney, S., Group, M., Langerod, A., Green, A., Provenzano, E., Wishart, G., Pinder, S., Watson, P., Markowetz, F., Murphy, L., Ellis, I., Purushotham, A., Borresen-Dale, A. L., Brenton, J. D., Tavare, S., Caldas, C., and Aparicio, S. (2012) The genomic and transcriptomic architecture of 2,000 breast tumours reveals novel subgroups. *Nature* 486, 346-352
11. Chen, X., Leung, S. Y., Yuen, S. T., Chu, K. M., Ji, J., Li, R., Chan, A. S., Law, S., Troyanskaya, O. G., Wong, J., So, S., Botstein, D., and Brown, P. O. (2003) Variation in gene expression patterns in human gastric cancers. *Mol. Biol. Cell* 14, 3208-3215
12. Yoshihara, K., Tajima, A., Komata, D., Yamamoto, T., Kodama, S., Fujiwara, H., Suzuki, M., Onishi, Y., Hatae, M., Sueyoshi, K., Fujiwara, H., Kudo, Y., Inoue, I., and Tanaka, K. (2009) Gene expression profiling of advanced-stage serous ovarian cancers distinguishes novel subclasses and implicates ZEB2 in tumor progression and prognosis. *Cancer Sci.* 100, 1421-1428
